# Cellular adaptations impact the biological activity of naphthalene diimide G-quadruplex ligands in ALT-positive osteosarcoma cells

**DOI:** 10.1038/s41419-025-07908-2

**Published:** 2025-08-01

**Authors:** Joanna Bidzinska, Lorenzo Di Pietro, Eisa Naghshineh, Cecilia Pandini, Filippo Doria, Nadia Zaffaroni, Paolo Gandellini, Stephen Neidle, Marco Folini

**Affiliations:** 1 Second Department of Radiology, Medical University of Gdańsk, Gdańsk, Poland; 2 Molecular Pharmacology Unit, Department of Experimental Oncology, Fondazione IRCCS Istituto Nazionale dei Tumori di Milano, Milan, Italy; 3 Department of Biosciences, University of Milan, Milan, Italy; 4 Department of Chemistry, University of Pavia, Pavia, Italy; 5 School of Pharmacy, University College London, London, UK

**Keywords:** Bone cancer, Preclinical research, Telomeres

## Abstract

Telomeric G-quadruplexes (G4s) represent intriguing targets for tumours characterized by the Alternative Lengthening of Telomere (ALT) mechanism. Here we have investigated the effects of two naphthalene diimide (NDI)-based G4 interacting agents (NMe2 and QN-302) in a pair of ALT-positive human osteosarcoma (U-2 OS and Saos-2) cell lines. Both NDIs displayed marked cell growth inhibitory activity associated with the induction of telomere dysfunctions. Moreover, NDI-treated cells were characterized by perturbations at the mitochondrial level as suggested by an increase in the production of reactive oxygen species, the occurrence of changes in mitochondria density and morphology. However, upon initial inhibition of cell growth, U-2 OS cells withstood ligand-induced stress compared to Saos-2 cells. This ability was in part sustained, in a ligand-dependent manner, by the lack of ALT activity inhibition, as indicated by the levels of telomeric C-circle DNA and of Bloom helicase, a member of the RecQ family of helicases. Moreover, marked basal antioxidant capacity, together with the capability to mount an antioxidant response that is in part mediated by the nuclear factor erythroid 2-related factor, has endowed U-2 OS cells with the ability to adapt to NDI exposure. Our data indicate that NDIs rapidly affect the growth of ALT cancer cells by interfering with telomere and mitochondria homeostasis and suggest that small molecule-mediated stabilization of G4s may be a promising therapeutic strategy in ALT-positive tumors. Nonetheless, depending on the individual NDI and the cell’s genetic background, cellular adjustment mechanisms may become activated. This, in turn may impinge on the biological activity of G4 interacting agents. Deciphering these mechanisms and the associated molecular determinants will help accelerating the development of G4-based therapeutic interventions in ALT tumors.

## Introduction

Human cancers display well-defined hallmarks that establish the molecular and biological framework of tumour cells and may serve as sources of biomarkers and/or therapeutic targets [1]. Among these common features, the limitless proliferative potential of cancer cells is accomplished by the maintenance of telomere length and integrity [2].

Telomeres are nucleoprotein structures located at the ends of linear chromosomes [3, 4]. They guarantee genome integrity by protecting chromosome ends from being recognized as sites of DNA damage and unwanted DNA repair [3]. Moreover, telomeres act as biological clocks by imposing a limit (the Hayflick limit) to the lifespan of normal cells [3] that lose telomeric DNA (nearly 70 bp/year in humans [5]) with each cell division cycle due to the “end replication problem” [6]. Critically shortened telomeres become dysfunctional and elicit a DNA damage response (DDR) which in turn results in cell cycle arrest and ultimately in the onset of replicative senescence or apoptosis [6]. Cancer cells may activate a telomere maintenance mechanism (TMM) in order to bypass such a tumour suppressor barrier and enable replicative immortality.

The reactivation of telomerase is the most common TMM in human cancers [7–9]. Several strategies aimed to interfere with telomerase expression and function for therapeutic purposes have been widely documented in different preclinical models of human tumours [10], leading the first-in-class telomerase inhibitor Imetelstat to enter clinical trials and being approved for medical use by the FDA in 2024 [11].

Nonetheless, nearly 10–15% of human tumours rely on the homologous recombination (HR)-based alternative lengthening of telomere (ALT) mechanism [9, 12]. The frequent activation of ALT in tumours of mesenchymal origin and its possible association with cancer aggressiveness [13] has made it an intriguing candidate as a therapeutic target for these malignancies [10, 13]. This hypothesis has been further supported by the assumption that tumours relying on telomerase to maintain telomeres could become resistant to telomerase inhibitors due to the emergence of ALT as an adaptive mechanism [14]. However, the fragmentary knowledge of factors controlling ALT in human tumours has thus far hindered the development of genuine ALT-targeting therapies [10, 13].

G-quadruplexes (G4s) are higher-order structures that may form within guanine rich nucleic acid sequences [15] comprising short repetitive G-tracts. G4s comprise a core of two or more stacked G-quartets (the square planar arrangement of four guanine residues) held together by intervening loops [15]. Telomeres have been the first biologically relevant G4 forming sequences to be studied in detail [16]. It was initially proposed that telomeric G4 could act as a barrier for the recombination events that sustain the ALT pathway [17]. More recently, it has been reported that the stabilization of G4s by small molecules (G4 ligands, G4Ls) may lead to an hyper-ALT phenotype [12, 18], as revealed by the increase in ALT-associated features (e.g., increased level of extrachromosomal telomeric circular DNA consequent to G4L-indcuced stalling of the replication fork [16, 19]). Also, impaired processing of HR intermediates is associated with increased levels of telomere replicative stress, thus leading ALT-positive tumour cells to apoptosis [12, 18, 20].

In the present study, the biological effects of telomeric G4 stabilization by G4Ls has been explored in a pair of ALT-positive osteosarcoma cell lines (U-2 OS/p53^WT^ and Saos-2/p53^del^) that represent the gold standard for telomerase-negative/ALT-positive cancer cells [21]. In particular, we focused on the compounds NMe2 [22, 23] and the novel experimental drug QN-302 (previously known as SOP1812) [24–27] - which has recently entered phase I clinical trials for solid tumours [28]. Both ligands belong to the most promising family of G4Ls characterized by a naphthalene diimide (NDI) core [28, 29] and have been previously reported to act as a telomeric G4L and to exert potent cytotoxic activity in telomerase-positive cancer cells [22–27].

## Materials and methods

### Cell lines and compounds

Human osteosarcoma cells (U-2 OS, HTB-96™; Saos-2, HTB-85™) were obtained from ATCC (LGC Standards, Milano, Italy). Cells were grown as monolayers and were cultured at 37 °C in a humidified 5% CO_2_ atmosphere, using McCoy’s 5 A Medium Modified (EuroClone S.p.A., Pero, Italy) supplemented with 10% (U-2 OS) or 15% (Saos-2) foetal bovine serum (EuroClone S.p.A). Cells were monitored by STR profiling (Cogentech, Milano, Italy) and checked for the lack of Mycoplasma contamination (MycoAlert^TM^ Mycoplasma Detection Kit, Lonza Walkersville Inc., Walkersville, MD), periodically.

The compounds NMe2 and QN-302 (SOP1812) had been synthesised as previously described [22–25]. The compounds were dissolved in DMSO (Merck Life Science S.r.l., Milano, Italy) to obtain a 10 mM stock solution, kept at −20 °C and diluted at the appropriate working concentrations immediately before use. N-acetyl-L-cysteine (NAC; A9165, Sigma-Aldrich, Saint Louis, MO) was prepared as 1 M solution every time before use, according to the provider’s instructions and filter-sterilized through 0.22 µm pore size Millex-GS filter (Millipore, Merck Life Science S.r.l.).

Target-specific siRNAs designed to silence the expression of *TP53* (sip53; sc-29435); *CDKN1A* (sip21; sc-44214), *BLM* (siBLM; sc-29808) and *NFE2L2* (siNrf2; sc-44332) as well as a control siRNA (siCTR; sc-37007) were purchased from Santa Cruz Biotechnology (Dallas, TX). siRNAs were dissolved in RNase-free water to make a 10 μM stock solution, stored at −20 °C and diluted to obtain a final concentration of 50 nM immediately before use.

### Cell-based experiments

To assess the effect of the G4Ls on U-2 OS and Saos-2 cell growth, cells were seeded at the appropriate density in 6-well plates and exposed to the indicated increasing concentrations of freshly dissolved compounds. Adherent cells were trypsinised and counted in a particle counter (Coulter Counter, Coulter Electronics, Luton, UK) after 2 days of G4L exposure. The compound concentrations inhibiting cell growth by 50% (IC_50_) and 80% (IC_80_) were calculated from the dose-response curves (% inhibition of cell growth with respect to untreated cells plotted as a function of the Log_10_ of compound concentrations) using GraphPad Prism 10.4.

To evaluate the effect of the G4Ls on cell growth over time, cells were seeded at the appropriate density in 75-cm^2^ tissue culture flasks and allowed to attach for 24 h. On the next day, U-2 OS and Saos-2 cells were treated with equitoxic amounts of the G4Ls (IC_50_ at day 2). Adherent cells were then harvested at the indicated time points and counted in a particle counter. Cell pellets of detached cells were prepared for subsequent analyses.

For transfection procedures, cells seeded at the appropriate density and allowed to attach for 24 h were incubated with Lipofectamine-2000^TM^ (Thermo Fisher Scientific Inc., Monza, Italy) in Opti-MEM I (Thermo Fisher Scientific Inc.) serum-free medium. After 15 min at room temperature, lipid-treated cells were added with Opti-MEM I medium containing the siRNAs at the final concentrations of 50 nM and incubated for 5 h at 37 °C. Cells were then harvested according to the timeline of each experiment and subsequently analysed. For the time-lapse assessment of cell growth, cells were seeded at the appropriate density in a 24-well plate and allowed to attach for 24 h. Transfection was carried out as described above followed by a 2-h exposure (pulse) to a subtoxic amount ( < 20% growth inhibition) of either G4Ls. Cells were then imaged (16 field/well; 4 well/sample) every 4 h up to 96 h by the Incucyte® SX5 Live-Cell Imaging and Analysis System (Sartorius Italy S.r.l., Varedo, Italy). Cell growth was analysed by Incucyte® image analysis software (Sartorius). Data have been reported as percent confluence from phase images upon normalization using GraphPad Prism 10.4.

For combination studies, NMe2 and ML216 (HY-12342; D.B.A. Italia, Segrate, Italy) were combined at fixed ratio in three concentrations (0.0625, 0.125, 0.25 μM and 7.5, 15 and 30 μM, respectively) and incubated for 2 days at 37 °C, 5% CO_2_. Cell growth inhibitory activity was assayed as described above. The type of pharmacological interaction was assessed by calculating the combination index (CI) at doses effecting 30%, 50%, 75% and 90% reduction of cell growth, according to the Chou and Talalay’s method [30] using the CompuSyn.Ink program.

### C-circle assay (CCA)

Telomeric C-circle DNA levels were assessed by a qPCR-based CCA [31], with minor modifications. Briefly, 32 ng of genomic DNA, obtained with the Qiagen Blood & Cell Culture DNA Midi Kit (Qiagen, Hilden, Germany) according to the manufacturer’s instructions, was incubated at 30 °C for 16 h in the presence (φ29 + ) or absence (φ29–) of φ29 DNA polymerase (2.5 U; New England BioLabs, Ipswich, MA). Upon heat inactivation at 70 °C for 20 min, reaction products were diluted to 0.4 ng/μL in 10 mM Tris pH 7.6 and subjected to qPCR amplification (QuantStudio 12 K Flex; Thermo Fisher Scientific Inc.) using QuantiTect SYBR Green PCR Kit (#204143; Qiagen), according to the following conditions: 95 °C for 15 min followed by 33 cycles amplification [95 °C, 15 s; 58 °C, 20 s] for telomeric DNA and 95°C for 5 min followed by 40 cycles amplification [95 °C, 15 s; 58 °C, 30 s] for *36B4* single copy gene. Primer pairs were as follow: Telomere forward (5′-CGGTTTGTTTGGGTTTGGGTTTGGGTTTGGGTTTGGGTT-3′), telomere reverse (5′-GGCTTGCCTTACCCTTACCCTTACCCTTACCCTTACCCT-3′); 36B4 forward (5′-CAGCAAGTGGGAAGGTGTAATCC-3′), 36B4 reverse (5′-CCATTCTATCATCAACGGGTACAA-3′) [31]. All primers were used at 500 nM final concentration.

Telomeric and *36B4* single copy gene (SCG) contents were obtained by interpolating threshold cycles (C_T_) to standard curves, as described in [31]. Telomeric content was normalized to SCG content and CC levels were calculated for each sample as CCA score by subtracting the normalized telomeric content of the φ29− control to the normalized telomeric content of the respective φ29+ sample. Total DNA from the telomerase-positive HeLa cells was used as a negative control.

### Assessment of the antioxidant response

Basal and G4L-modulated total antioxidant capacity was assessed by the OxiSelect™ Total Antioxidant Capacity (TAC) Assay Kit (STA-360, Cell Biolabs, Inc., San Diego, CA), according to the manufacturer’s instructions. The TAC of each sample has been determined by comparing the net OD_490_ values of the tested samples (20 μg protein lysate) to those of a uric acid standard curve. The data have been reported as μM Copper Reducing Equivalents (μM CRE) by converting the determined mM uric acid equivalents using the conversion factor of 2189 μM Cu^+^^+^/mM uric acid, accordingly to the manufacturer’s instruction.

The TransAM® NRF2 DNA-binding ELISA kit (#50296, Active Motif®, Carlsbad, CA) was used to monitor Nrf2 binding activity. Briefly, nuclear extracts were obtained from untreated and G4L-treated cells by Nuclear Extract Kit (#40010, Active Motif®) according to the manufacturer’s protocol and quantified by standard methods. Nuclear extracts (10 μL; 1.0 μg/μL) diluted in lysis buffer were dispensed in each well of a 96-well plate containing immobilized oligonucleotide bearing the ARE (antioxidant responsive element) consensus binding site (5´-GTCACAGTGACTCAGCAGAATCTG-3´). The Nrf2 primary antibody (1:1 000 dilution in 1 × Antibody Binding Buffer), which recognizes an epitope on Nrf2 protein upon DNA binding, was added to each well. To monitor the specificity of the assay, a wild-type or mutated consensus oligonucleotide was added to each well prior to addition of the nuclear extracts, according to the manufacturer’s instructions. The plate was incubated for 1 h at room temperature and after washing in 1 × washing buffer ( × 3 times) it was probed for 1 h at room temperature with HRP-linked secondary antibody (1:1000). A positive nuclear extract control (5 µg) provided with the kit was used as a control for Nrf2 activation. After washing in 1 × washing buffer ( × 4 times), 100 µl of developing solution was added to all wells and the plate incubated for 15 min at room temperature protected from direct light. After adding 100 µl of stop solution the absorbance was read on a microplate reader at 450 nm with a reference wavelength of 655 nm. Data have been reported as OD_450_, according to the manufacturer’s instructions.

### Real-time RT-qPCR

Total RNA was obtained from U-2 OS and Saos-2 cells using the RNeasy Plus Mini Kit (Qiagen). Randomly primed total RNA was reverse transcribed using the High-Capacity RNA-to-cDNA™ Kit (Applied Biosystems, Carlsbad, CA, USA) and gene expression levels were analysed by the specific NQO1 TaqMan® Gene expression assay (Hs01045993_g1; Applied Biosystems).

The amplification reactions were run on QuantStudio™ 12 K Flex Real-Time PCR System (Thermo Fisher) under the following conditions: one cycle at 95 °C for 20 s and then 1 s at 95 °C, 20 s at 60 °C for 40 cycles. The mRNA expression levels in the different samples were reported either as 2^−ΔCt^ or 2^−ΔΔCt^. *GAPDH* (Hs02786624_g1; Applied Biosystems) was used as normalizer.

### Statistical analyses

Statistical analyses were performed using GraphPad Prism 10.4. The sample sizes required for the experiments were estimated based on preliminary results. If not otherwise specified, data were obtained from at least three independent experiments and have been reported as individual data points alongside mean ± s.d. The two-tailed, non-parametric test and unpaired t-test were used to analyse differences between samples. Two-way ANOVA followed by Bonferroni post-test was performed for multiple comparisons between groups. *p* < 0.05 were considered statistically significant.

Additional Materials and Methods and the original scans of the western immunoblots presented in this study have been reported in the Supplementary Information.

## Results

### The exposure of ALT-positive osteosarcoma cells to G4Ls remarkably elicits telomere dysfunctions

A 2-day exposure of U-2 OS and Saos-2 cells to increasing concentrations of G4Ls resulted in a marked, dose-dependent inhibition of cell growth (Supplementary Fig. S1), with IC_50_ and IC_80_ values falling within the sub-micromolar range (Table 1). Furthermore, QN-302 demonstrated superior growth inhibitory activity over NMe2, as evidenced by lower IC_50_ and IC_80_ values at day 2 for both cell lines (Table 1).

**Table 1:** Concentrations of G4Ls inhibiting osteosarcoma cell growth.

	IC_50_ (nM)		IC_80_ (nM)	
	NMe2	QN-302	NMe2	QN-302
U-2 OS	230 ± 20	16 ± 1	610 ± 30	64 ± 9
Saos-2	410 ± 30	43 ± 2	1600 ± 80	282 ± 21

Concentrations of G4Ls inhibiting 50% (IC_50_) and 80% (IC_80_) of cell growth obtained after a 2-day exposure to the indicated compounds.

**Fig. 1:**
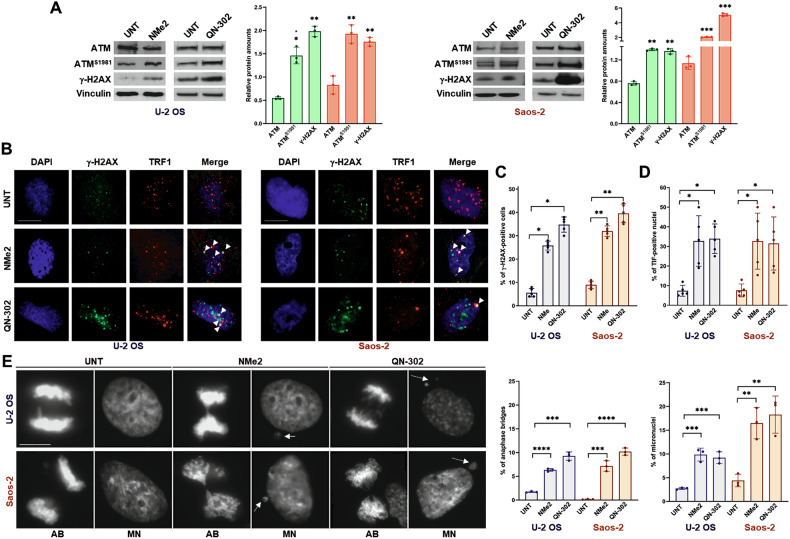
The exposure of ALT-positive osteosarcoma cells to G4Ls elicited DNA damage associated to telomere dysfunctions. **A** Representative western immunoblotting showing the amount of proteins involved in the signalling of DNA damage assessed in untreated U-2 OS and Saos-2 cells and after a 2-day exposure to an equitoxic amount (IC_50_) of NMe2 (green) or QN-302 (red). Vinculin was used to ensure equal protein loading. Cropped images of selected proteins are shown. The graphs show the quantification of the relative protein abundance in treated *vs*. untreated cells upon normalization to Vinculin. Data have been reported as mean values ± s.d. (*N* = 3); *p < 0.05; **p < 0.01; ***p < 0.001 (two-tailed unpaired *t*-test); **B** Representative photomicrographs showing the immunofluorescence analysis of γ-H2AX (green) and TRF1 (red) in untreated (UNT) U-2 OS and Saos-2 cells and after a 2-day exposure to an equitoxic amount (IC_50_) of each G4L. Nuclei were counterstained with DAPI (blue). Arrows indicate the fluorescence foci (yellow) arising from the co-localization between the two proteins. Scale bar: 10 μm; magnification: × 60; **C** Quantification of DNA damage foci in untreated (UNT) and NMe2- or QN-302-treated U-2 OS and Saos-2 cells. Data have been reported as percentage of nuclei that stained positive for γ-H2AX (*N* = 5); *p < 0.05; **p < 0.01 (two-tailed Mann Whitney test); **D** Quantification of telomere dysfunction induced foci (TIF) in U-2 OS and Saos-2 cells after a 2-day exposure to an equitoxic amount (IC_50_) of NMe2 or QN-302. Cells with one or more γ-H2AX co-localized with TRF1 (yellow dots in **B**) were scored as TIF-positive cells. Data have been reported as the percentage of TIF-positive nuclei within the overall population of γ-H2AX–positive nuclei in treated and untreated (UNT) cells. Bars represent mean values ± s.d. (*N* = 5); *p < 0.05 (two-tailed Mann–Whitney test) (**E**) Representative photomicrographs showing anaphase bridges (AB) and micronuclei (MN, white arrows) in NMe2- and QN-302-treated compared to untreated U-2 OS and Saos-2 cells stained with DAPI. Scale bar: 10 μm; magnification: × 40. The graphs on the right reports the percentage of anaphase bridges and of micronuclei within the overall cell population in untreated (UNT) and NMe2- or QN-302 treated U-2 OS and Saos-2 cells. Data represent mean values ± s.d. (*N* = 3); **p < 0.01: ***p < 0.001; ****p < 0.001 (two-tailed unpaired *t*-test).

Additionally, a 2-day exposure of U-2 OS and Saos-2 cells to equitoxic doses (IC_50_ reported in Table 1) of the ligands induced a DNA damage response (DDR), as shown by increased levels of phospho-ATM (Ataxia-telangiectasia mutated) on Serine 1981 and of γ-H2AX (Fig. 1A). This was further confirmed by a marked increase in γ-H2AX nuclear foci in G4L-treated cells compared to untreated controls (Fig. 1B, C). Importantly, a significant proportion of these γ-H2AX foci were observed to localize at the telomeric region (Fig. 1B, D), as evidenced by the overlap between the γ-H2AX fluorescence signal and that of TRF1, a telomere-associated protein used as a marker for interphase telomeres [32, 33]. The co-localization of DNA damage with telomeric markers suggests a high prevalence of telomere-dysfunction-induced foci (TIFs), and thus, dysfunctional telomeres, in G4L-treated compared to untreated cells [32, 33]. Indeed, a significant increase in the percentage of mitotic cells exhibiting anaphase bridges, as well as cells with micronuclei, was observed in G4L-treated cells compared to untreated controls (Fig. 1E).

### OS cells are distinctly affected in their growing capabilities upon exposure to G4Ls

**Fig. 2:**
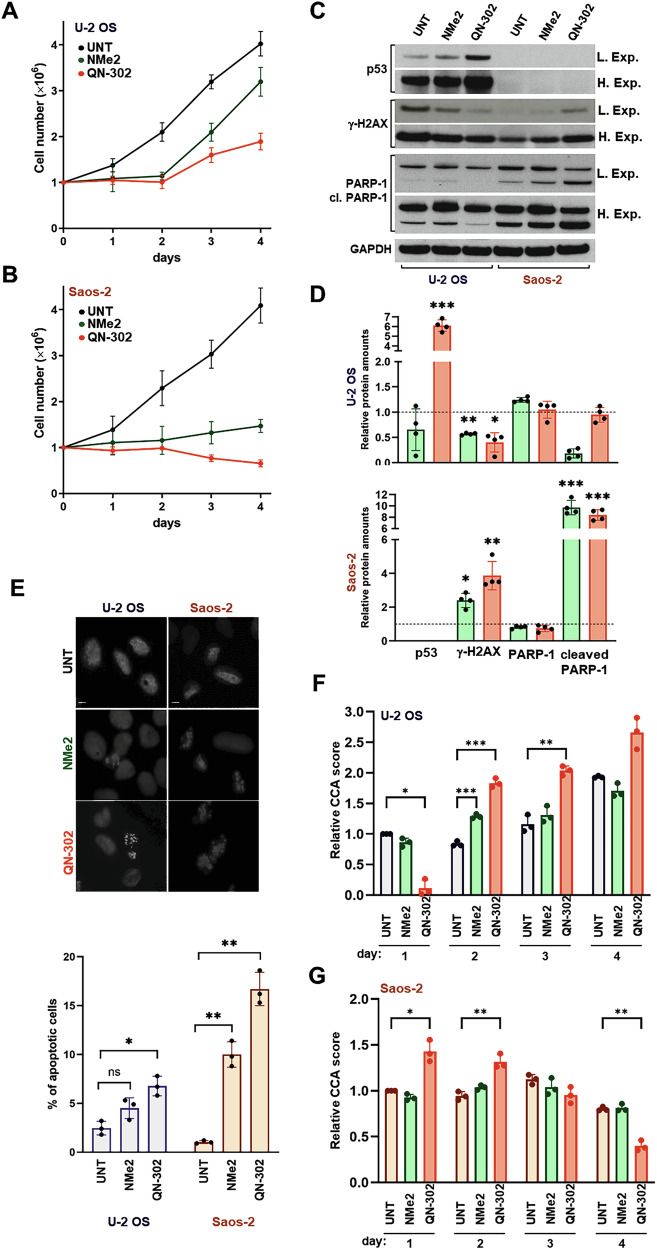
Osteosarcoma cells are distinctly affected in their growth capabilities upon exposure to the NDIs. Growth kinetics of untreated (black line), NMe2- (green line) and QN-302-treated (red line) U-2 OS (**A**) and Saos-2 (**B**) cells. Data have been reported as the number of growing cells as a function of days of treatment and represent mean values ± s.d. (*N* = 6); **C** Representative western immunoblotting showing the amount of proteins in U-2 OS and Saos-2 cells either untreated (UNT) and after a 3-day exposure to an equitoxic amount (IC_50_) of NMe2 or QN-302. GAPDH was used to ensure equal protein loading. Cropped images of selected proteins are shown. L. Exp.: low exposure; H. Exp.: High exposure; **D** Quantification of protein amounts in U-2 OS (*top panel*) and Saos-2 (*bottom panel*) cells after a 3-day exposure to equitoxic amounts of NMe2 (green bars) or QN-302 (red bars). Data have been reported as relative protein amounts with respect to untreated cells following normalization toward GAPDH and represents mean values ± s.d. (*N* = 4). Dotted horizontal line indicates the protein levels in untreated cells; *p < 0.05; **p < 0.01; ***p < 0.001 (two-tailed unpaired *t*-test); **E** Representative photomicrographs showing apoptotic nuclear morphology in NMe2- and QN-302-treated compared to untreated U-2 OS and Saos-2 cells stained with DAPI. Scale bar: 10 μm; magnification: × 20. The graph on the right reports the percentage of apoptotic cells within the overall cell population. Data represents mean values ± s.d. (*N* = 3); *p < 0.05; **p < 0.01 (two-tailed unpaired *t*-test); Quantification of telomeric C-circle DNA levels over time in untreated (UNT) U-2 OS (**F**) and Saos-2 (**G**) cells and after the exposure to equitoxic amounts (IC_50_) of the indicated G4Ls. Data have been reported as relative CCA score with respect to untreated cells at day 1 (*N* = 3); *p < 0.05; **p < 0.01; ***p < 0.001 (2-way ANOVA).

Although G4Ls exhibited comparable short-term biological activity, the analysis of cell growth kinetics revealed distinct effects on U-2 OS and Saos-2 cell growth after exposure to equitoxic amounts (IC_50_) of either ligand. Specifically, a progressive increase in the number of growing cells was observed in NMe2-treated and, to a lesser extent, QN-302-treated U-2 OS cells, following an initial impairment of cell growth within the first two days of exposure to each G4L (Fig. 2A). In contrast, the total number of Saos-2 cells remained stable or decreased over time upon exposure to equitoxic doses of NMe2 or QN-302 (Fig. 2B). In this context, compared to day 2 (Fig. 1A), U-2 OS cells treated with NMe2 showed a significant reduction in γ-H2AX levels relative to untreated cells (Fig. 2C, D), accompanied by no significant changes in p53 protein levels (Fig. 2C, D). These findings suggest that an efficient DNA repair mechanism may contribute to the resumption of U-2 OS cell growth following a 3-day exposure to NMe2. Similarly, compared to day 2 (Fig. 1A), QN-302-treated U-2 OS cells displayed significantly lower γ-H2AX levels on day 3 relative to untreated cells (Fig. 2C, D). Nonetheless, QN-302 exposure also led to a pronounced accumulation of p53 protein (Fig. 2C, D), which likely accounts for the reduced ability to resume cell growth relative to NMe2-treated U-2 OS cells (Fig. 2B).

In this regard, the observed increase in p53 protein in QN-302-treated U-2 OS cells is consistent with previous findings showing that the exposure of liposarcoma cells to QN-302 leads to a significant accumulation of p53 protein, likely due to the compound’s ability to interfere with MDM2 expression and affect the MDM2-p53 feedback loop [34]. Accordingly, in parallel with the accumulation of p53 protein, a large accumulation of MDM2 protein was also observed over time in U-2 OS but not in Saos-2 cells exposed to equitoxic amounts of QN-302 (Supplementary Figure S2A). Moreover, *TP53* silencing led to a slight but significant increase in the inhibition of U-2 OS cell growth induced by QN-302, whereas it had no effect on the activity of NMe2 (Supplementary Fig. S2B).

Conversely, after 3 days of exposure to the ligands, Saos-2 cells displayed increased γ-H2AX levels and evidence of PARP-1 cleavage (Fig. 2C, D), a biochemical marker of apoptosis. Notably, a significant higher percentage of apoptotic cells was observed in G4L-treated Saos-2 compared to U-2 OS cells (Fig. 2E), although untreated U-2 OS cells exhibited slightly higher levels of spontaneous apoptosis compared to untreated Saos-2 cells (Fig. 2E).

Furthermore, treatment of U-2 OS and Saos-2 cells with equitoxic amounts of NMe2 did not cause major modifications in the levels of telomeric C-circle (CC) DNA (Fig. 2F, G), except for a 1.5-fold increase appreciable at day 2 in NMe2-treated U-2 OS cells and with fluctuations at other time points comparable to those observed in untreated cells (Fig. 2F). Conversely, exposure of U-2 OS cells to an equitoxic dose of QN-302 resulted in a marked reduction in CC levels on day 1, followed by a significant and progressive increase at later time points compared to untreated cells (Fig. 2F). On the other hand, upon an initial increase in CC levels observed on days 1 and 2, a decrease at later time points, particularly after 4 days of drug exposure, was observed in Saos-2 cells exposed to an equitoxic amount of QN-302 (Fig. 2G), indicating impaired ALT activity over time.

### Bloom helicase helps U-2 OS cells to withstand G4L-induced telomeric stress

**Fig. 3:**
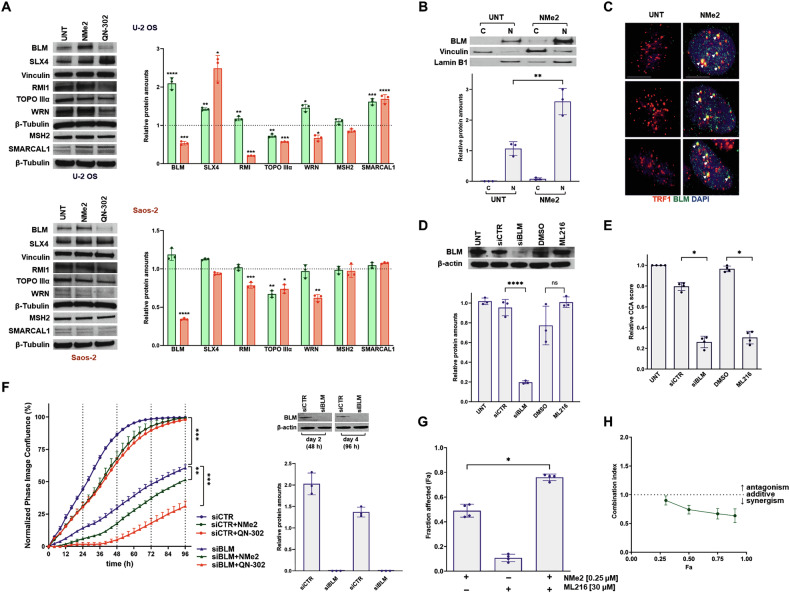
The BLM helicase may represent a determinant of U-2 OS cell response to NDI exposure. **A** Representative western immunoblotting showing the amount of the indicated proteins in U-2 OS (*top panel*) and Saos-2 (*bottom panel*) cells either untreated (UNT) and after a 2-day exposure to an equitoxic amount (IC_50_) of NMe2 or QN-302. Vinculin or β-tubulin were used to ensure equal protein loading. Cropped images of selected proteins are shown. The graphs on the side of each panel report the quantification of the protein amounts in treated *vs*. untreated cells following normalization with respect to vinculin or β-tubulin and represents mean values ± s.d. (N = 3); *p < 0.05; **p < 0.01; ****p < 0.0001 (two-tailed unpaired *t*-test); **B** Representative Western immunoblotting showing BLM protein amounts in the cytoplasmic (c) and nuclear (n) fractions of untreated U-2 OS cells and after a 2-day exposure to NMe2 (IC_50_). Lamin B1 and vinculin were used to ensure equal protein loading and proper nucleus/cytoplasm fractionation. Cropped images of selected proteins are shown. The graph on the bottom reports the quantification of the protein amounts. Data have been reported as relative protein amounts with respect to vinculin (cytoplasmic fraction) or lamin B1 (nuclear fraction) and represents mean values ± s.d. (*N* = 3); **p < 0.01 (two tailed unpaired *t*-test); **C** Representative photomicrographs showing the co-localization between BLM (green) and TRF1 (red) assessed by immunofluorescence in untreated U-2 OS cells and after a 2-day exposure to NMe2 (IC_50_). Nuclei were counterstained with DAPI. Arrows indicate the fluorescence foci (yellow) arising from the co-localization between the two proteins. Merged images are shown; scale bars: 10 μm; magnification: × 60; **D** Representative western immunoblotting showing the amount of BLM protein in non-transfected (UNT), siCTR- and siBLM-transfected as well as DMSO-exposed and ML216-treated U-2 OS cells. β-actin was used to ensure equal protein loading. Cropped images of selected proteins are shown. The graphs on the bottom reports the quantification of the protein amounts. Data have been reported as relative protein amounts with respect to β-actin and represents mean values ± s.d. (*N* = 3); ****p < 0.0001 (two tailed unpaired *t*-test); n.s.: not statistically significant. **E** Quantification of telomeric C-circle DNA levels in untreated (UNT), siCTR- and siBLM-transfected U-2 OS cells and after a 2-day exposure to 30 μM ML216. Data have been reported as relative CCA score in the indicated samples with respect to UNT cells and represent mean values ± s.d. (*N* = 4); *p < 0.05 (two-tailed Mann–Whitney test); **F** Assessment of cell growth kinetics in siCTR (•)- and siBLM (▲)-transfected U-2 OS cells either untreated (blue) or after a 2-h exposure (pulse) to subtoxic amounts of NMe2 (green) or QN-302 (red). Data have been reported as the percentage of phase image confluency (determined by Incucyte® SX5 Live-Cell Imaging and Analysis System) normalized to the first time point (T_0_) using the normalization function in GraphPad. Data represent mean values ± s.d. (*N* = 4); *p < 0.05; **p < 0.01; ***p < 0.001 (2-way ANOVA). The panel on the right reports a representative western immunoblotting showing BLM protein amounts in siCTR- and siBLM-transfected U-2- OS cells at the indicated time points. The graphs report the quantification of BLM protein amounts. Data have been reported as relative protein amounts with respect to β-actin and represents mean values ± s.d. (*N* = 3). **G** Quantification of cell growth inhibition in U-2 OS cells exposed for 2 days to NMe2 (0.25 μM) and ML216 (30 μM) alone or in combination. Data have been reported as the extent of cell growth inhibition (fraction affected) and represent mean values ± s.d. (*N* = 4); *p < 0.05 (two-tailed Mann–Whitney test); **H** Synergistic pharmacological interaction observed in U-2 OS cells after a 2-day exposure to the combination NMe2:ML216 (1:120) as indicated by the values of the combination index (C.I.) as a function of the fraction affected (Fa) calculated as previously reported [30].

The analysis of proteins known to play a role in the ALT mechanism [35] has revealed that the two cell lines displayed distinct profiles in the levels of ALT-associated factors following exposure to either ligand. Specifically, greater variations in the levels of ALT-associated proteins were clearly observed in U-2 OS cells compared to Saos-2 cells after treatment with either G4 ligand (Fig. 3A). These include SLX4 (a component of the SLX4-SLX1-ERCC4 complex which promotes the resolution of recombination intermediates [36]), RMI1 (that together with Bloom helicase (BLM) [37], and Topoisomerase IIIα, constitutes the BTR dissolvase complex required for ALT-mediated telomere synthesis [35, 36]), WRN (a member of the RecQ family of helicases [37], which is variably required for telomere maintenance in ALT-positive cells [38]), and SMARCAL1 (a SWI/SNF-related factor that has an important function in preventing telomere replication stress [39]), but not of MSH2 (a component of the mismatch repair factor MutSβ that may prevent the accumulation of telomeric G4 structures [40]). Notably, a two-fold increase in BLM protein was observed in U-2 OS cells following a 2-day exposure to NMe2 at its IC_50_ concentration (Fig. 3A), whereas only a mild and not statistically significant increase was detected in Saos-2 cells exposed to an equitoxic dose of the same compound (Fig. 3A). Conversely, BLM protein levels were significantly downregulated in both cell lines treated with equal active amounts of QN-302 (Fig. 3A).

It has been reported that BLM plays a central role both in ALT-dependent telomere maintenance [36, 41, 42] and HR-directed DNA repair [42–44]. In this regard, the nuclei of NMe2-treated U-2 OS cells were characterized by markedly higher amounts of BLM protein compared to untreated cells (Fig. 3B, C), a fraction of which was found to localize at telomeres, as indicated by the overlap between BLM foci (green fluorescence) and TRF1 (red fluorescence) (Fig. 3C).

Moreover, siRNA-mediated silencing of BLM resulted in a pronounced decrease of BLM protein levels in U-2 OS cells (Fig. 3D), which was associated with a significant reduction in CC levels (Fig. 3E). Similarly, treatment with the specific inhibitor (ML216, [45]) of BLM activity also led to reduced CC levels (Fig. 3E), although BLM protein levels remained unaffected compared to controls (Fig. 3D).

Notably, BLM silencing significantly impaired U-2 OS cell growth over time (Fig. 3F). However, transfection with BLM siRNA prior to a 2-h pulse exposure to a subtoxic concentration of NMe2 (<20% growth inhibition) enhanced the compound’s growth-inhibitory effect compared to control siRNA-transfected cells (Fig. 3F). These findings were corroborated by the observation that combined treatment of U-2 OS cells with ML216 and NMe2 led to a synergistic interaction, as shown by greater cell growth inhibition compared to either agent alone (Fig. 3G; Supplementary Fig. S3), and by the trend of the calculated combination index relative to the fraction of affected cells (Fig. 3H). Altogether, these data suggest that BLM plays a critical role in mediating the response of U-2 OS cells to G4L exposure. Supporting this, reduced BLM protein amounts were detected in cells exposed to an equitoxic amount of QN-302 (Fig. 3A), which has superior cell growth inhibitory activity compared to NMe2 (Table 1). Additionally, BLM silencing prior to QN-302 treatment resulted in a significant enhancement of cell growth inhibitory activity compared to BLM proficient (siCTR-transfected) U-2 OS cells (Fig. 3F). However, under the same experimental conditions, we found that BLM silencing had only a mild effect on the growth of Saos-2 cells and did not enhance the activity of QN-302 (Supplementary Fig. S4). This did result in the enhancement of NMe2 cell growth inhibitory activity, likely due to the compound’s ability to induce a slight increase in BLM protein levels (Fig. 3A).

### An antioxidant response may contribute to the ability of osteosarcoma cells to cope with G4L exposure

**Fig. 4:**
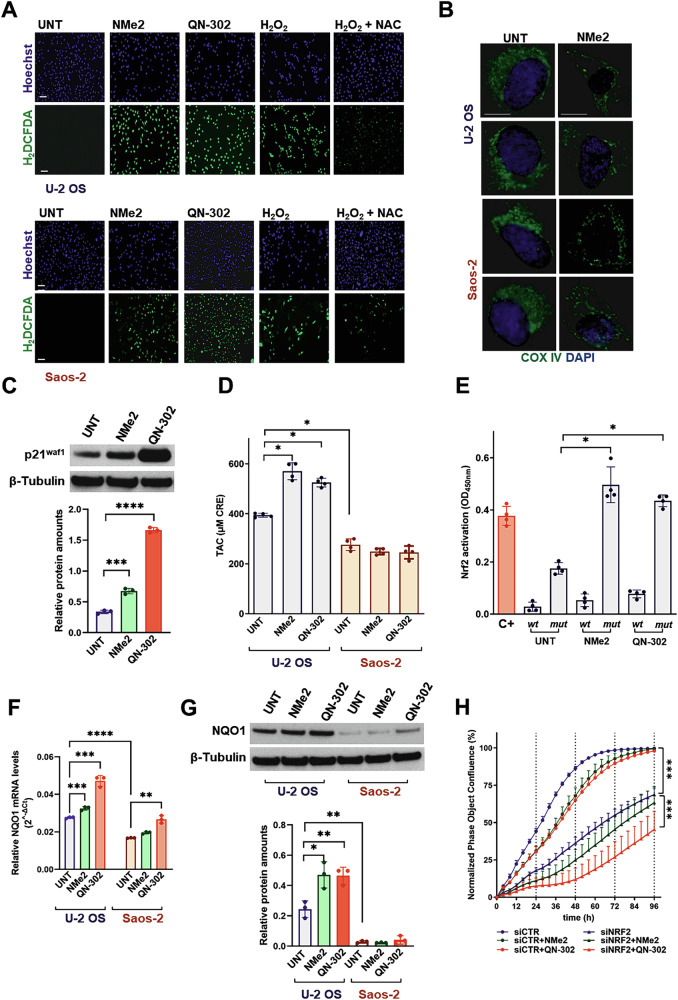
Assessment of ROS and mitochondrial dysfunctions as well as of the antioxidant response in osteosarcoma cells upon exposure to G4Ls. **A** Representative photomicrographs showing the detection of ROS upon H_2_DCFDA staining of living U-2 OS and Saos-2 cells both untreated (UNT) and after a 1-day exposure to NMe2 or QN-302. Hydrogen peroxide (H_2_O_2_) in the absence or presence of N-acetylcysteine (NAC) was used as positive and negative controls for ROS detection, respectively. Nuclei were counterstained with Hoechst 3342. Scale bars: 100 μm; magnification: × 10; **B** Representative photomicrographs showing mitochondrial morphological alterations in NMe2-treated vs. untreated U-2 OS and Saos-2 cells probed with an anti-COX IV antibody (green). Nuclei were counterstained with DAPI. Merged images are shown; scale bar: 10 μm; magnification: × 60; **C** Representative western immunoblotting showing p21^Waf1^ protein amounts in untreated U-2 OS cells and after a 2-day exposure to either G4L (IC_50_). β-tubulin was used to ensure equal protein loading. Cropped images of selected proteins are shown. The graph on the bottom reports the quantification of p21^Waf1^ protein amounts in the indicated samples. Data have been reported as relative protein amounts with respect to β-tubulin and represents mean values ± s.d. (*N* = 3). ***p < 0.001; ****p < 0.0001 (two tailed unpaired *t*-test); **D** Quantifica*t*ion of the total antioxidant capacity (TAC) in untreated (UNT) U-2 OS and Saos-2 cells and after a 2-day exposure to equitoxic (IC_50_) amounts of NMe2 or QN-302. Data have been reported as the total antioxidant capacity, expressed as μM copper-reducing equivalents (μM CRE), according to the manufacturer’s instructions. Bars represent mean values ± s.d. (*N* = 4); *p < 0.05 (two-tailed Mann-Whitney test); **E** Quantification of the Nrf2 binding activity to ARE sequences in untreated U-2 OS cells and after a 2-day exposure to either G4L (IC_50_). Data have been reported as OD read at 450 nm in tested samples in the presence of wild-type (wt) or mutated (mut) ARE-containing consensus sequence used to test for binding competition. C + : internal positive control for Nrf2 binding activity provided with the kit. Bars represent mean values ± s.d. (*N* = 4); *p < 0.05 (two-tailed Mann–Whitney test); **F** Assessment of *NQO1* mRNA levels in untreated U-2 OS and Saos-2 cells and after a 2-day exposure to either G4L (IC_50_). RT-qPCR data were reported as 2^−ΔCt^ and represents mean values ± s.d. (*N* = 3). **p < 0.01; ***p < 0.001; ****p < 0.0001 (two tailed unpaired *t*-test). *GAPDH* was used as normalizer; **G** Representative western immunoblotting showing the amounts of the Nrf2 downstream target NQO1 in untreated U-2 OS and Saos-2 cells and after a 2-day exposure to either G4L (IC_50_). β-tubulin was used to ensure equal protein loading. Cropped images of selected proteins are shown. The graph at the bottom reports the quantification of NQO1 protein amounts in the indicated samples. Data have been reported as relative protein amounts with respect to β-tubulin and represents mean values ± s.d. (*N* = 3). *p < 0.05; **p < 0.01 (two tailed unpaired *t*-test); **H** Assessment of cell growth kinetics in siCTR (•)- and siNrf2 (▲)-transfected U-2 OS cells either untreated (blue) or after a 2-h exposure (pulse) to subtoxic amounts of NMe2 (green) or QN-302 (red). Data have been reported as the percentage of phase image confluency (determined by Incucyte® SX5 Live-Cell Imaging and Analysis System) normalized to the first time point (T_0_) using the normalization function in GraphPad and represent mean values ± s.d. (*N* = 4); ***p < 0.001 (2-way ANOVA).

It has been reported that the high basal levels of DNA damage that are characteristic of ALT cancer cells [46] are associated with mitochondrial dysfunctions and reactive oxygen species (ROS) production [47, 48]. To examine this concept, fluorescence microscopy analysis of G4L-treated U-2 OS and Saos-2 cells revealed a marked staining with the ROS detector H_2_DFCDA compared to untreated cells or to cells treated with hydrogen peroxide ± N-acetylcysteine (Fig. 4A). Moreover, a pronounced impairment in the density and morphology of mitochondria was also observed as highlighted by the reduced intensity and the peculiar distribution pattern of the fluorescent signal observed in G4L-treated compared to untreated cells probed with an anti-COX IV antibody (Fig. 4B; Supplementary Fig. S5A) or stained with a mitochondria-specific dye (Supplementary Fig. S5B).

It has been established that mitochondrial dysfunctions and ROS production consequent to telomere–dependent and –independent DNA damage may lead to the up-regulation of p21^Waf1^ [49] as an antioxidant defensive mechanism to promote cell survival under oxidative stress [50]. Both G4Ls produced an accumulation of p21^Waf1^ in U-2 OS (Fig. 4C) but not in Saos-2 cells (Supplementary Fig. S5C). Notably, silencing of p21^Waf1^ significantly impaired the growth of U-2 OS cells over time (Supplementary Fig. S5D) and resulted in the enhancement of NMe2 and QN-302 growth-inhibitory effects after a 2-h pulse exposure to a subtoxic concentration of either ligand ( < 20% growth inhibition) compared to control siRNA-transfected cells (Supplementary Fig. S5D).

The accumulation of p21^Waf1^ as a possible antioxidant defensive mechanism is in keeping with the observation that U-2 OS cells were characterized by higher basal levels of total antioxidant activity (TAC) than Saos-2 cells (Fig. 4D) and that a significant increase in TAC was appreciable in G4L-treated U-2 OS cells, whereas no changes in treatment-induced compared to basal TAC levels were appreciable in Saos-2 cells exposed to equitoxic amounts of G4L (Fig. 4D).

P21^Waf1^-dependent cell survival under oxidative stress may be mediated in part by the activation of the nuclear factor erythroid 2-related factor (Nrf2) [50], which relocates to the nucleus where it binds to ARE sequences and regulates the transcription of genes involved in the antioxidant response [51]. Indeed, a nearly 3-fold increase in the ARE binding activity of Nrf2 was observed in G4L-treated compared to untreated U-2 OS cells (Fig. 4E), whereas no appreciable enrichment in the binding of Nrf2 was detected in G4L-treated compared to untreated Saos-2 cells (Supplementary Fig. S5E). This result was mirrored by the markedly higher mRNA and protein amounts of NAD(P)H quinone dehydrogenase 1 (NQO1), a Nrf2 downstream factor observed in G4L-treated compared to untreated U-2 OS cells (Fig. 4F, G). Conversely, Saos-2 cells showed markedly lower basal levels of NQO1 (Fig. 4F, G), the amounts of which were negligibly affected upon ligand exposure compared to U-2 OS cells (Fig. 4F, G). This suggests that U-2 OS cells can mount an antioxidant response, at least in part mediated by Nrf2, upon exposure to G4 ligands. In line with this, Nrf2 silencing led to a marked reduction in Nrf2 binding activity to ARE sequences compared to siCTR- or non-transfected cells (Supplementary Fig. S6A). Additionally, it resulted in decreased basal and ligand-induced levels of NQO1 mRNA and protein (Supplementary Fig. S6B, C). Notably, transfection with siNrf2 (Supplementary Fig. S6D) significantly impaired U-2 OS cell growth over time (Fig. 4H). However, Nrf2 silencing prior to a 2-h pulse exposure to subtoxic concentrations ( < 20% growth inhibition) of either ligand enhanced the cell growth-inhibitory effects of the G4Ls, with the effect being particularly pronounced for QN-302, compared to cells transfected with control siRNA (Fig. 4H). Moreover, although Saos-2 cells do not appear to mount an antioxidant response upon exposure to G4Ls, Nrf2 silencing prior to a 2-h pulse with subtoxic amounts of either ligand led to an increase in the cell growth-inhibitory activity of both NMe2 and QN-302 compared to siCTR-treated cells (Supplementary Figure S7). This may be consistent with the role of Nrf2 in regulating redox homeostasis under physiological conditions [51].

## Discussion

The search for ALT-selective cancer therapies has been challenged by the fragmentary information currently available on specific factors involved in the engagement and fuelling of this telomere maintenance mechanism in human tumours [12, 13]. Nonetheless, some progress has been made in the attempt to characterize ALT at the molecular level which have resulted in some strategies being tested at preclinical level as potential ALT-directed therapeutic interventions [9, 10, 13, 20]. These include the search for potential synthetic lethal interactions by exploiting ALT-associated genetic alterations (e.g., *ATRX*/*DAXX*) or the high level of telomeric DNA damage that characterizes ALT-positive cells (e.g., ATR inhibitors); the disruption of APBs or APB-associated factors (e.g., TSPYL5 depletion); the inhibition or depletion of HR-associated factors and the induction of ALT hyperactivation upon inhibition of FANCM (Fanconi anaemia complementation group M) helicase or through the stabilization of telomeric G4s [9, 10, 12, 13, 20].

We report here that the biological effects exerted by G4 interacting agents are dependent on the nature and potency of the G4L as well as on the genetic background of the ALT-positive osteosarcoma cell lines. Thus, short-term (2-day) exposure of U-2 OS and Saos-2 cells to either ligand resulted in significant growth inhibition and a notable increase in TIFs, anaphase bridges, and micronuclei, that are features typically associated not only with genomic instability but also with the presence of dysfunctional telomeres [52, 53]. Nonetheless, compared to Saos-2 cells, which are characterized by the deletion of *TP53* [54] and exhibit growth inhibition over time and apoptosis induction upon ligand exposure, U-2 OS cells were able to tolerate ligand treatment. This is shown by the full or partial recovery of cell growth over time in U-2 OS cells treated with equitoxic doses of NMe2 or QN-302. This recovery was accompanied by a reduction in γ-H2AX levels and the absence of PARP-1 cleavage, suggesting an ability to repair DNA damage following treatment. Furthermore, U-2 OS cells exposed to NMe2 showed reduced p53 protein levels, whereas treatment with an equitoxic dose of QN-302 resulted in a marked accumulation of p53. This observation is consistent with recent findings showing that the exposure of patient-derived dedifferentiated liposarcoma cell lines to QN-302 leads to substantial p53 accumulation, due to the compound’s ability to directly interfere with MDM2 expression [34]. Silencing of *TP53* in U-2 OS cells led to a slight but significant increase in the growth inhibition induced by QN-302, while, as expected, it had no effect on the activity of NMe2.

We also observed that the two ligands interfered differently with the ALT mechanism, as assessed by the production of telomeric CC DNA, which is currently considered to be a reproducible marker of ALT activity [55, 56]. Thus, despite the differential effect on cell growth observed in the two osteosarcoma cell lines, NMe2 did not appear to significantly interfere with CC production, as their levels were similar in treated and untreated cells, apart from a transient increase observed in U-2 OS cells on day 2 of treated compared to untreated cells. In contrast, following a significant reduction in CC in U-2 OS cells after 1 day of treatment with QN-302, a progressive increase in CC levels over time was observed, suggesting a possible stimulation of the ALT mechanism [19]. Conversely, in Saos-2 cells, there was an approximately 1.5-fold increase in CC levels on days 1 and 2, followed by a gradual decrease after treatment with QN-302, suggesting a possible inhibition of the ALT mechanism in dying cells. These observations are consistent with literature data showing that the growth inhibition of U-2 OS and Saos-2 cells treated with the telomeric G4 ligand RHPS4 was not associated with the inhibition of the ALT mechanism, but rather with an increase in markers associated with this mechanism, including elevated levels of CC [19]. These findings highlight how the ALT mechanism may play an ambiguous role following the occurrence of replicative stress at telomeric level. In fact, it has been reported that the transient activation of the ALT mechanism, in the absence of growth inhibition or cell death induction, may represent a protective response to stress, including oxidative stress [57]. Moreover, it has been demonstrated that ALT activation can ultimately serve as a pathway leading to cell death following treatment with G4 ligands [18]. These can cause protein trapping on DNA, thus leading to replication fork stalling and ALT induction [58].

Similarly, the two cell lines responded differently to treatment with NMe2 and QN-302 in terms of modulation of protein factors known to play a role in ALT [35–38]. Specifically, with the exception of a significant reduction in BLM observed in both cell lines treated with QN-302, larger variations in the levels of ALT-associated proteins such as SLX4, RMI1, WRN, and SMARCAL1 were evident in U-2 OS cells compared to Saos-2 cells following treatment with either ligand. Given that the exact mechanism underlying the biogenesis of ALT-associated CC is still not fully understood, how the distinct patterns of ALT-associated factors correlate with the observed CC profiles in the two osteosarcoma cell lines treated with G4Ls remains an aspect that warrants further in-depth investigation.

Nonetheless, a significant increase in BLM protein amounts was observed in U-2 OS cells, and, to only a very small extent, in Saos-2 cells exposed to NMe2. In this regard, BLM silencing resulted in the significant impairment of U-2 OS cell growth, as well as a marked enhancement of the cell growth inhibitory activity of both ligands. Furthermore, pharmacological inhibition of BLM activity produced a synergistic interaction with NMe2. It has been reported that BLM may act as a determinant of the response to different DNA-damaging agents. Specifically, BLM inhibition has been shown to enhance the cytotoxic activity of alkylating agents in multiple myeloma and prostate cancer cells [43, 59], and it may offer therapeutic benefit in tumours that are intrinsically resistant to PARP inhibitors or radiation [60].

The hypothesis that BLM could be a determinant of the response to DNA interaction agents, including certain G4Ls, is indirectly supported by our finding that reduced BLM levels were observed in cells exposed to QN-302, a compound that exerts greater growth inhibitory activity than NMe2. This aligns with the evidence that the distinct pattern of gene down-regulation induced by QN-302 in cancer cells contributes to its superior efficacy compared to other closely related NDI-based G4Ls [61].

Our data showed that the exposure of osteosarcoma cells to G4L may result in mitochondrial dysfunction and ROS production. In this regard, it has been reported that the baseline levels of mitochondrial defects typically associated with ALT cells become increasingly apparent in the setting of induced telomeric DNA damage [48] as well as that a self-amplifying cycle may occur between telomere-localized DNA damage and ROS generation [62]. These interconnected events likely reflect the genotoxic stress accompanying the relatively low efficiency with which ALT cells maintain and/or replicate telomeres compared to telomerase-positive cancer cells [47]. Notably, it has been recently reported that cellular ROS are important causative factors in the evolution of ALT-mediated telomere maintenance in ATRX-deficient cancers [63]. Nonetheless, we cannot exclude the possibility that the observed mitochondrial perturbations could be the consequence of ligand-mediated direct stabilization of mitochondrial DNA G4 [64]. However, we found that the total antioxidant capacity and the capability to mount an antioxidant adaptive response, in part mediated by Nrf2, may enable osteosarcoma cells to withstand G4L-mediated inhibition of cell growth in a cell-type dependent manner. It has been demonstrated that the activation of the Nrf2 pathway provides a survival advantage in response to selective pressure (e.g., lack of nutrients, oxidative stress, hypoxia) during tumour development and may confer resistance to chemotherapy or radiation during tumour progression [51, 65]. However, under our experimental conditions, silencing of Nrf2 only partly enhanced NMe2-mediated inhibition of cell growth. This evidence is in accord with the observation that antioxidant cell defences may be fostered through a feed-forward loop involving the de novo translation of Nrf2 protein in the presence of persistent oxidative stress [66] and supports the uncertainty that still surrounds Nrf2 as a possible target in cancer [51, 65]. Moreover, we cannot exclude the possibility that additional antioxidant defences (e.g., ferroptosis inhibition [67]) or other adaptive responses (e.g., autophagy induction, phenotype switching [68–70]) may endow U-2 OS cells with the ability to alleviate G4L-induced stress. In this regard and in line with data reported here, we previously reported that RNAi-mediated depletion of p21^Waf1^ enabled melanoma cells to overcome protective autophagy induced by the exposure to an anthracene-based G4L [69].

In conclusion, small molecule-mediated G4 targeting may represent a promising therapeutic strategy for ALT-positive osteosarcoma and for other tumours that maintain telomeres *via* ALT (e.g., soft-tissue sarcomas) [13]. A wide variety of small molecules have been evaluated for their G4 stabilizing properties in preclinical models of human cancer [15, 71]. However, CX-5461 and QN-302 are the only G4Ls currently being evaluated in clinical trials for cancer patients [28, 71], indicating that the identification of clinically relevant G4Ls may not be a trivial activity [68]. In this regard, it has been reported that cell-specific G4 landscapes [34] as well as the enrichment of G4 structures alongside associated transcription factors may be the determinants of cell-specific transcriptional programs and consequently influence the biological activity of G4Ls [72]. Moreover, the need for unification and standardization of the experimental settings in living systems alongside the elucidation of the adaptive responses to G4Ls both as a function of the tested ligand and the cell genetic backgrounds [15, 68] remain imperatives in order to draw meaningful conclusions on the antitumor properties of these compounds in human patients. In addition, the identification of the molecular determinants underlying the different cellular responses to G4Ls is of paramount importance to accelerate the development of G4-based therapeutic interventions and help G4Ls to be developed into clinically useful therapeutic agents.

## Supplementary information

Supplementary Materials and Methods

Supplementary Figures

Original scans for western immunoblotting

## Data Availability

All data generated or analyzed during this study are included in this published article and its supplementary information files. Public data sharing is not applicable to this article as no datasets were generated or analyzed during the current study.
