# Supplementary material for: Cellular adaptations impact the biological activity of naphthalene diimide G-quadruplex ligands in ALT-positive osteosarcoma cells
Source: Cell Death Dis. 2025 Aug 1;16(1):581. doi: 10.1038/s41419-025-07908-2 (PMC12316980; doi:10.1038/s41419-025-07908-2)
Supplement: Supplementary file 1 — Supplementary Materials and Methods [file 41419_2025_7908_MOESM1_ESM.pdf]

Supplementary Information

Cellular Adaptations Impact the Biological Activity of Naphthalene Diimide G-quadruplex Ligands in ALT-positive Osteosarcoma Cells

Joanna Bidzinska[1*], Lorenzo Di Pietro[2*], Eisa Naghshineh[2*], Cecilia Pandini[3], Filippo Doria[4], Nadia Zaffaroni[2], Paolo Gandellini[3], Stephen Neidle[5], Marco Folini[2]

[1]Second Department of Radiology, Medical University of Gdańsk, Smoluchowskiego 17 Str., 80-214 Gdańsk, Poland;

[2]Molecular Pharmacology Unit, Department of Experimental Oncology, Fondazione IRCCS Istituto Nazionale dei Tumori di Milano, Via G.A. Amadeo 42, 20133 Milan, Italy;

[3]Department of Biosciences, University of Milan, Via Celoria 26, 20133 Milan, Italy

[4]Department of Chemistry, University of Pavia, V.le Taramelli 10, 27100 Pavia, Italy.

[5]School of Pharmacy, University College London, London WC1N 1AX, United Kingdom

*These authors contributed equally to this work

Corresponding author: Dr. Marco Folini, marco.folini@istitutotumori.mi.it.

Supplementary Materials and Methods

Primary antibodies

The following primary antibodies have been used for western immunoblotting or immunofluorescence analyses.

Antibody name	Part No.	Company	Dilution
Anti-ATM	ab78	Abcam (Prodotti Gianni Srl, Milano Italy)	1:1 000
Anti-β-Actin	Ab8226	Abcam	1:2 000
Anti-β-Tubulin	Ab6046	Abcam	1:500
Anti-BLM	ab2179	Abcam	1:2 000
Anti-COX IV	ab16056	Abcam	1:300
Anti-Lamin B1	ab16048	Abcam	1:1 000
Anti-MSH2	Sc-376384	Santa Cruz Biotechnology (Dallas, TX)	1:300
Anti-NQO1	#3187	Cell Signaling Technology (Danvers, MA)	1:1 000
Anti-p21/WAF1 (F-5)	sc-6246	Santa Cruz Biotechnology	1:200
Anti-p53 (DO-1)	sc-126	Santa Cruz Biotechnology	1:500
Anti-PARP	#9542	Cell Signaling Technology	1:1 000
Anti-Phospho-Histone H2AX (S139)	ab11174	Cell Signaling Technolgy	1:300 (IF),<br>1:1 000 (WB)
Anti-Phospho-ATM (Ser1981)	ab81292	Abcam	1:1 000
Anti-RMI1	NB100-1720	Novus Biologicals (Bio-Techne Srl, Italy)	1:500
Anti-SLX4	NBP1-28679	Novus Biologicals	1:2 000
Anti-SMARCAL1	A301-616A	Bethyl Laboratories (CliniSciences, Italy)	1:2 000
Anti-Topoisomerase IIIα (N-20)	sc-11257	Santa Cruz Biotechnology	1:200
Anti-TRF1	ab10579	Abcam	1:250
Anti-Vinculin	V9131	Merck Life Science Srl (Milano, Italy)	1:5 000
Anti-WRN	A-300-239A	Bethyl Laboratories	1:5 000

Western immunoblotting

Twenty-five micrograms of total protein extracts prepared according to standard methods was fractioned by SDS-PAGE (NuPAGE, Thermo Fisher Scientific Inc., Monza, Italy) and transferred onto Hybond nitrocellulose filters (GE Healthcare Life Sciences, Buckinghamshire, UK). When needed, protein extract was fractionated with the use of Nuclear/Cytosol Fractionation Kit (JM-K266-26, MBL International Corporation, Woburn, MA) and 10 μg of protein extracts was used for SDS-PAGE. Filters were blocked for 1 h at room temperature in 1× PBS-Tween20, 5% skim milk or in 5% bovine serum albumin and then incubated overnight at 4 °C with primary antibodies. The filters were then probed with secondary, HRP-linked whole antibodies (GE Healthcare) for 1 h at room temperature and blotted proteins detected using Novex® ECL HRP Chemiluminescent detection system (Thermo Fisher Scientific Inc.). Filters were then subjected to autoradiography. The films were scanned and acquired images were subjected to densitometric analysis using ImageJ 1.46r. If not otherwise specified, protein quantification has been reported with respect to untreated cells as mean values ± s.d. from at least three independent experiments.

Fluorescence microscopy analyses

Osteosarcoma cells (1.0×10^5 cells) were grown onto glass coverslips, fixed with 4% paraformaldehyde/PBS for 15 minutes at room temperature and permeabilized with ice-cold 1:1 methanol/acetone solution for 15 minutes. Cells were then probed with the desired primary antibody at appropriate dilution for 1 h at room temperature and subsequently incubated with AlexaFluor®488 or AlexaFluor®594 (Thermo Fisher Scientific Inc.) secondary antibody of the appropriate epitope. Glass slides were then mounted using Fluoroshield mounting medium with 4′6′-diamidino-2-phenylindole (DAPI) (ab104139, Abcam) for nuclei counterstaining. To label mitochondria, MitoTracker® Green FM (M7514, Thermo Fisher Scientific Inc.) was used according to the manufacturer's instructions. Images were acquired (magnification ×60 or ×100) using a Nikon Eclipse E600 fluorescence microscope (Nikon, Tokyo, Japan) using the NIS-Elements software

(Nikon) and processed by Adobe Photoshop Image Reader 7.0 or with ImageJ 1.46r. The number of γ-H2AX and telomere dysfunction induced (TIF) nuclear foci, anaphase bridges (quantified by identifying mitotic figures in anaphase with a filamentous connection linking two aligned plates), micronuclei and apoptotic cells was assessed by two independent observers on 200 cells in at least three different microscopy fields from three independent experiments. Cells with one or more γ-H2AX co-localized with TRF1 were scored as TIF-positive cells, as described in [1].
The detection of ROS was performed by Image-IT™ LIVE Green Reactive Oxygen Species Detection Kit (I36007; Thermo Fisher Scientific Inc.), according to the manufacturer's instructions. Briefly, cells were seeded at the appropriate density and incubated at 37°C in a humidified 5% CO2 atmosphere. On the next day, NMe2, QN-302, H2O2 (100 μM ± 10mM N-acetyl-L-cysteine) were added to the medium, separately or together, at the desired working concentrations. After 24 h cells were exposed to 10 μM of 5-(and-6)-carboxy-2',7'-dichlorodihydrofluorescein diacetate (carboxy-H_2DCFDA) for 30 minutes at 37ºC, following the manufacturer's recommendations. During the last 5 min of incubation, 1 μg/mL of Hoechst 3342 dye solution (#62249, Thermo Fisher Scientific Inc.) was added for live-cell fluorescent nuclear staining. Images were acquired immediately using a Nikon Eclipse E600 microscope (magnification ×10) using the NIS-Elements software and processed as described above.

[1] Zheng XH, Nie X, Fang Y, Zhang Z, Xiao Y, Mao Z, et al. A Cisplatin Derivative Tetra-Pt(bpy) as an Oncotherapeutic Agent for Targeting ALT Cancer. J Natl Cancer Inst. 109, djx061 (2017).
