# Supplementary material for: Cellular adaptations impact the biological activity of naphthalene diimide G-quadruplex ligands in ALT-positive osteosarcoma cells
Source: Cell Death Dis. 2025 Aug 1;16(1):581. doi: 10.1038/s41419-025-07908-2 (PMC12316980; doi:10.1038/s41419-025-07908-2)
Supplement: Supplementary file 2 — Supplementary Figures [file 41419_2025_7908_MOESM2_ESM.pdf]

Supplementary Figures

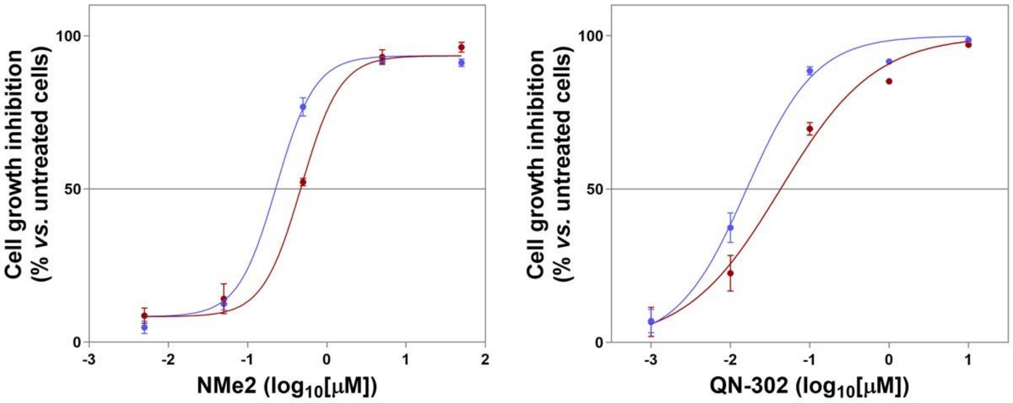

Supplementary Figure S1. Dose-response curves obtained after a 2-day exposure of U-2 OS (blue) and Saos-2 (red) cells to increasing concentrations of NMe2 (left) or QN-302 (right). Data have been reported as the percentage of cell growth inhibition with respect to untreated cells as a function of the Log_{10} compound concentration (μM) using the log(inhibitor) *vs.* normalized response function in GraphPad Prism 10.4 and represent mean values ± s.d. (N=6).

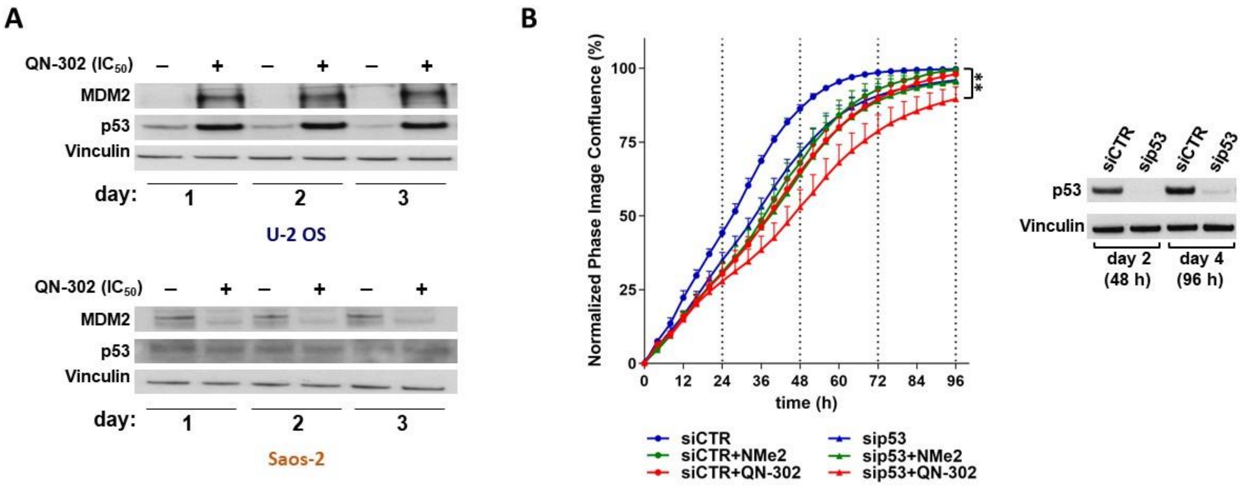

Supplementary Figure S2. (**A**) Representative western immunoblotting showing the amounts of MDM2 and p53 proteins over time in U-2 OS and Saos-2 cells either untreated (–) and after the exposure (+) to an equitoxic amount of QN-302 (IC_{50} at 48 h). Cropped images of selected proteins are shown. Vinculin was used to ensure for equal protein loading; (**B**) Assessment of cell growth kinetics in siCTR (•)- and sip53 (▲)-transfected U-2 OS cells either untreated (blue) or after a 2-h exposure (pulse) to subtoxic amounts of NMe2 (green) or QN-302 (red). Data have been reported as the percentage of phase image confluency (determined by Incucyte® SX5 Live-Cell Imaging and Analysis System) normalized to the first time point (T_0) using the normalization function in GraphPad. Data represent mean values ± s.d. (N=4); $**p < 0.01$ (2-way ANOVA). The panel on the right reports a representative western immunoblotting showing p53 protein abundance in siCTR- and sip53-transfected U-2 OS cells at the indicated time points.

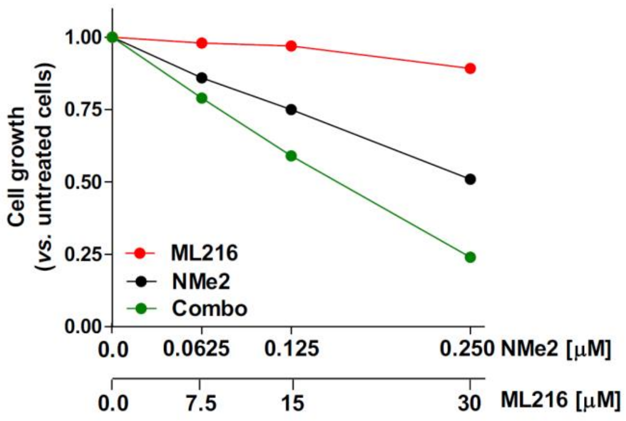

Supplementary Figure S3. Dose-response curves of U-2 OS cells incubated for 2 days in the presence of NMe2 (●), ML216 (●) or both compounds (●) administered at fixed ratio in three concentrations. Data have been reported as the percentage of cell growth with respect to untreated cells and represent mean values (N=4).

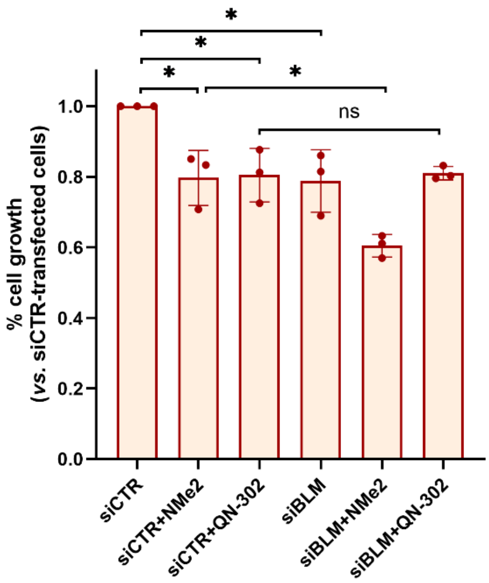

Supplementary Figure S4. Cell growth of siCTR- and siBLM-transfected Saos-2 cells either untreated or after a 2-h exposure (pulse) to subtoxic amounts (~IC_{20}) of NMe2 or QN-302. Data have been reported as the percentage of growing cells with respect to untreated siCTR-transfected cells at day 2 after treatment and represent mean values ± s.d. (N=3); *p < 0.05 (unpaired t-test); ns: not statistically significant.

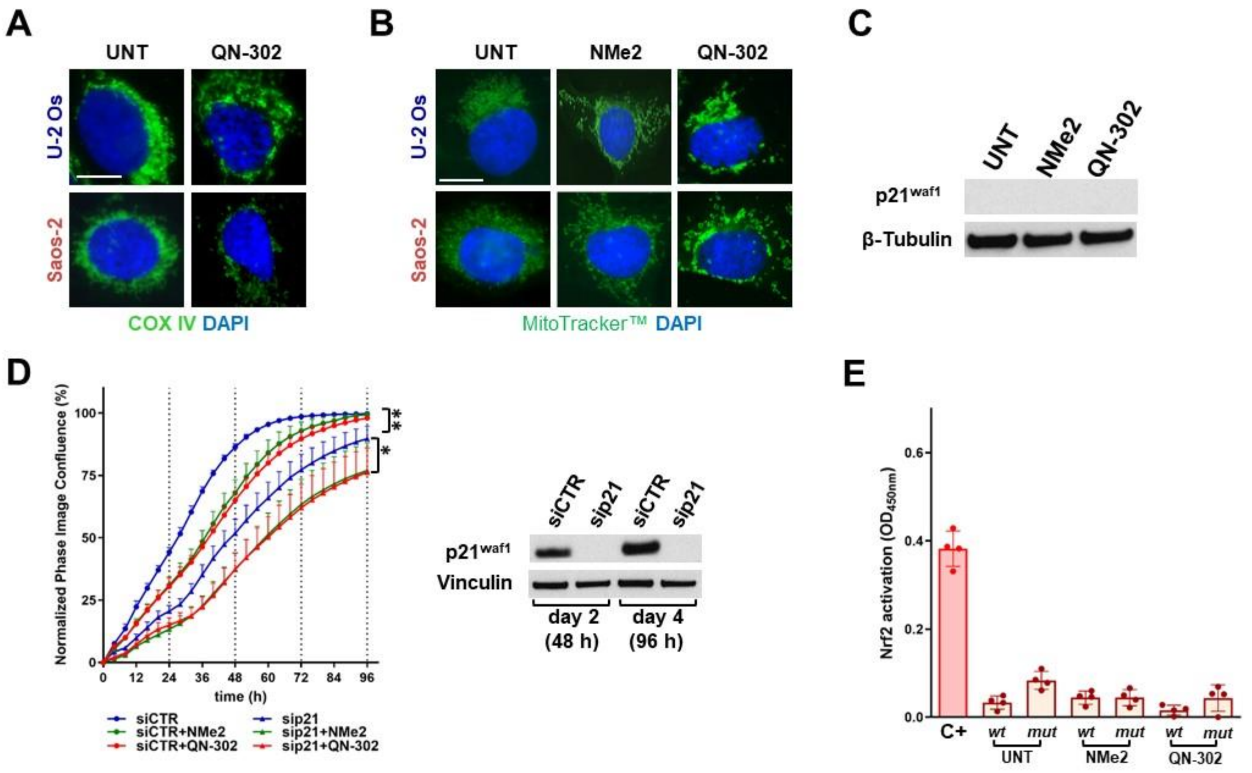

Supplementary Figure S5. (**A**) Representative photomicrographs showing mitochondrial morphological alterations in QN-302-treated *vs.* untreated U-2 OS and Saos-2 cells probed with an anti-COX IV antibody (green). Nuclei were counterstained with DAPI. Merged images are shown; scale bar: 10 µm; magnification: ×60; (**B**) Representative photomicrographs showing mitochondrial morphological alterations in NMe2- or QN-302-treated *vs.* untreated (UNT) U-2 OS and Saos-2 cells probed with MitoTracker™ dye (green). Nuclei were counterstained with DAPI. Merged images are shown; scale bar: 10 µm; magnification: ×60; (**C**) Representative $p21^{Waf1}$ western immunoblotting in untreated Saos-2 cells and after a 2-day exposure to the indicated G4L (IC_{50}). β-tubulin was used to ensure equal protein loading. Cropped images of selected proteins are shown; (**D**) Assessment of cell growth kinetics in siCTR (•)- and sip21 (▲)-transfected U-2 OS cells either untreated (blue) or after a 2-h exposure (pulse) to subtoxic amounts of NMe2 (green) or QN-302 (red). Data have been reported as the percentage of phase image confluency (determined by Incucyte® SX5 Live-Cell Imaging and Analysis System) normalized to the first time point (T_0) using the normalization function in GraphPad. Data represent mean values ± s.d. (N=4); *p < 0.05; **p < 0.01 (2-way ANOVA). The panel on the right reports a representative western immunoblotting showing $p21^{Waf1}$ protein amounts in siCTR- and sip21-transfected U-2 OS cells at the indicated time points; (**E**) Quantification of the Nrf2 binding activity to ARE sequences in untreated Saos-2 cells and after a 2-day exposure to either G4L (IC_{50}). Data have been reported as OD read at 450 nm in tested samples in the presence of wild-type (wt) or mutated (mut) ARE-containing consensus sequence used to test for binding competition. C+: internal positive control for Nrf2 binding activity provided with the kit. Bars represent mean values ± s.d. (N=4).

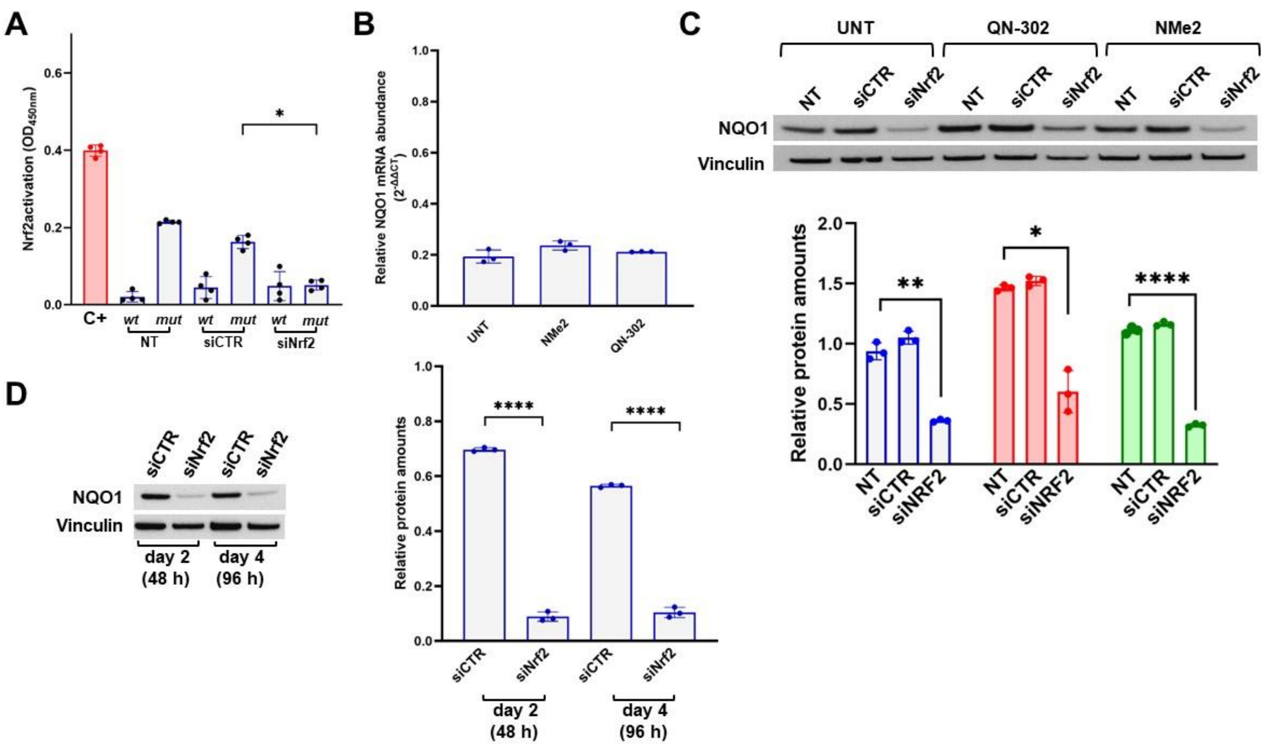

Supplementary Figure S6. (**A**) Quantification of the Nrf2 binding activity to ARE sequences in non-transfected (NT), siCTR- and siNrf2-transfected U-2 OS cells. Data have been reported as OD read at 450 nm in tested samples in the presence of wild-type (wt) or mutated (mut) ARE-containing consensus sequence used to test for binding competition. C+: internal positive control for Nrf2 binding activity provided with the kit. Bars represent mean values ± s.d. (N=4); **p* < 0.05 (two-tailed Mann-Whitney test); (**B**) Relative NQO1 mRNA abundance in siNrf2- *vs.* siCTR-transfected U-2 OS cells either untreated or after a 2-day exposure to equitoxic amounts (IC_{50}) of NMe2 or QN-302. Data have been reported as $2^{-\Delta\Delta Ct}$ in siNrf2 *vs.* siCTR-transfected cells after normalization toward *GAPDH* and represent mean values ± s.d. (N=3); (**C**) Representative western immunoblotting showing NQO1 protein amounts in non-transfected (NT), siCTR- and siNrf2-transfected U-2 OS cells, untreated and after a 2-day exposure to the indicated G4L (IC_{50}). Vinculin was used to ensure equal protein loading. Cropped images of selected proteins are shown. The graph at the bottom reports the quantification of NQO1 protein amounts in the indicated samples. Data have been reported as relative protein amounts with respect to vinculin and represents mean values ± s.d. (N=3); **p* < 0.05; ***p* < 0.01; *****p* < 0.0001 (two tailed unpaired *t*-test); (**D**) Representative western immunoblotting showing NQO1 protein amounts in siCTR- and siNrf2-transfected U-2 OS cells at the indicated time points. The graph on the right reports the quantification of NQO1 protein amounts in the indicated samples. Data have been reported as relative protein amounts with respect to vinculin and represents mean values ± s.d. (N=3); *****p* < 0.0001 (two tailed unpaired *t*-test).

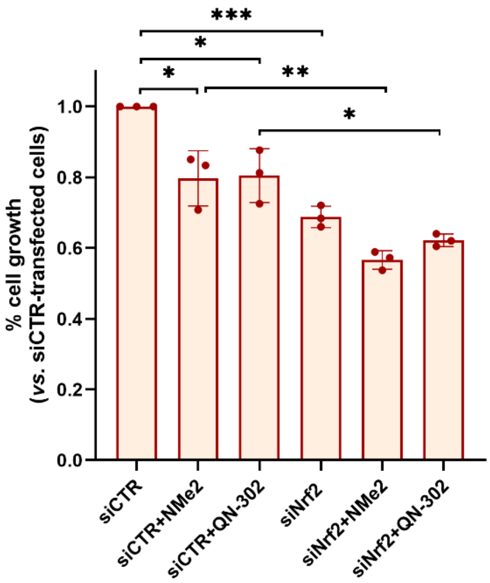

Supplementary Figure S7. Cell growth of siCTR- and siNrf2-transfected Saos-2 cells either untreated or after a 2-h exposure (pulse) to subtoxic amounts of NMe2 or QN-302. Data have been reported as the percentage of growing cells with respect to untreated siCTR-transfected cells at day 2 after treatment and represent mean values ± s.d. (N=3); **p* < 0.05 (unpaired *t*-test); ns: not statistically significant.
