# Supplementary material for: Cellular adaptations impact the biological activity of naphthalene diimide G-quadruplex ligands in ALT-positive osteosarcoma cells
Source: Cell Death Dis. 2025 Aug 1;16(1):581. doi: 10.1038/s41419-025-07908-2 (PMC12316980; doi:10.1038/s41419-025-07908-2)
Supplement: Supplementary file 3 — Original scans for western immunoblotting [file 41419_2025_7908_MOESM3_ESM.pdf]

FIG 1A

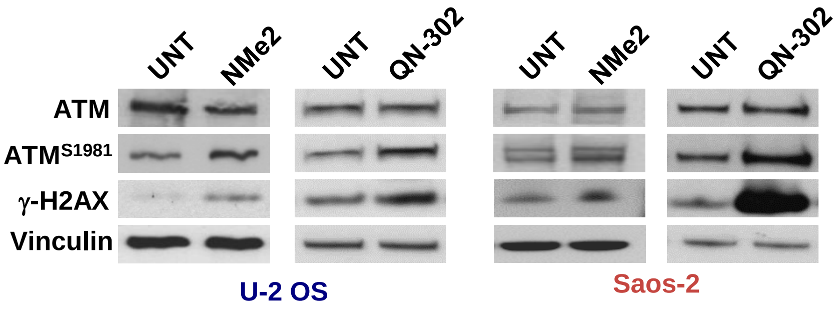

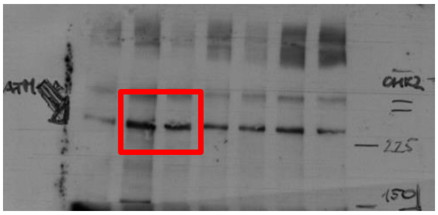

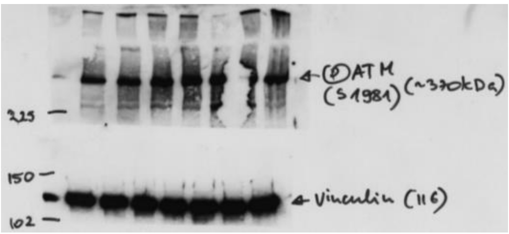

high exposure

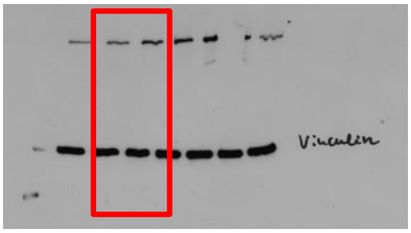

Low exposure

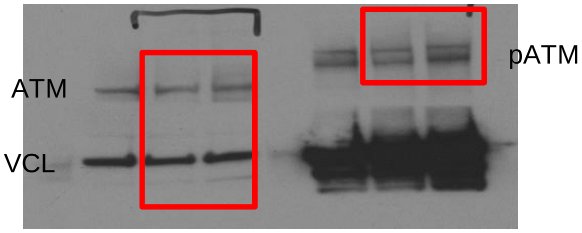

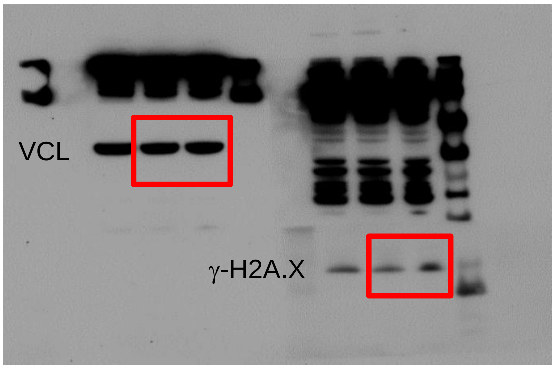

Saos-2/NMe2

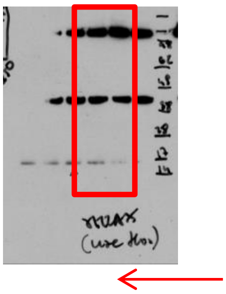

U-2 OS/NMe2

FIG 1A

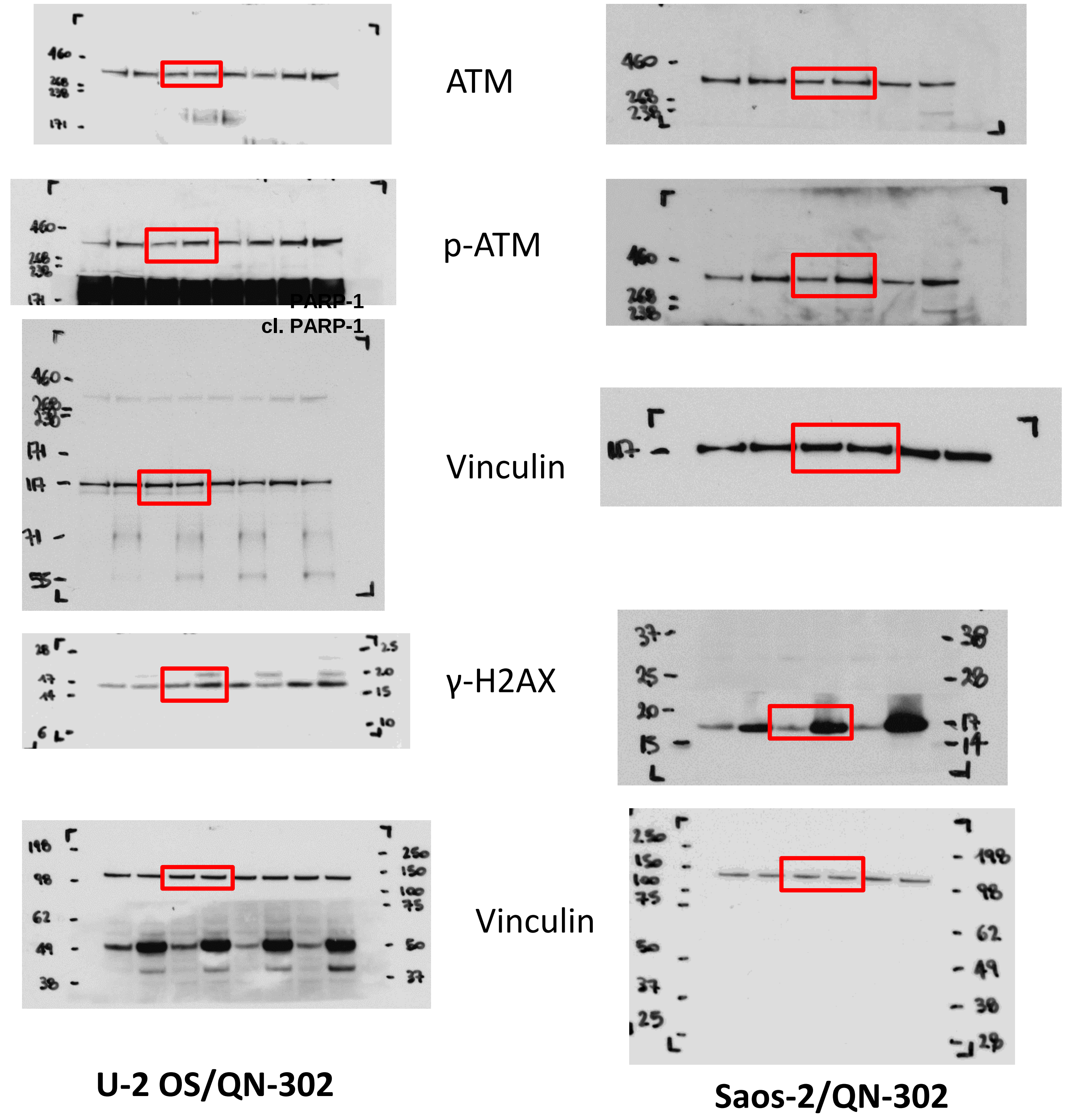

ATM
p-ATM
PARP-1
cl. PARP-1
Vinculin
γ-H2AX
Vinculin
U-2 OS/QN-302
Saos-2/QN-302

Figure 2C

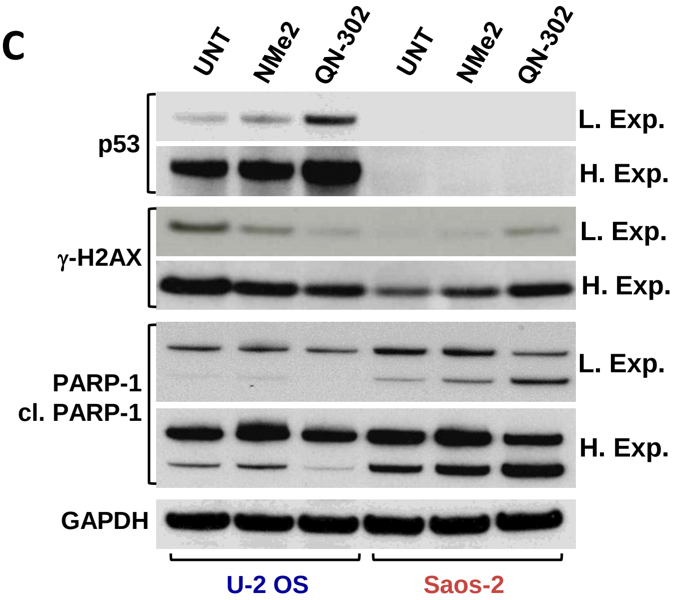

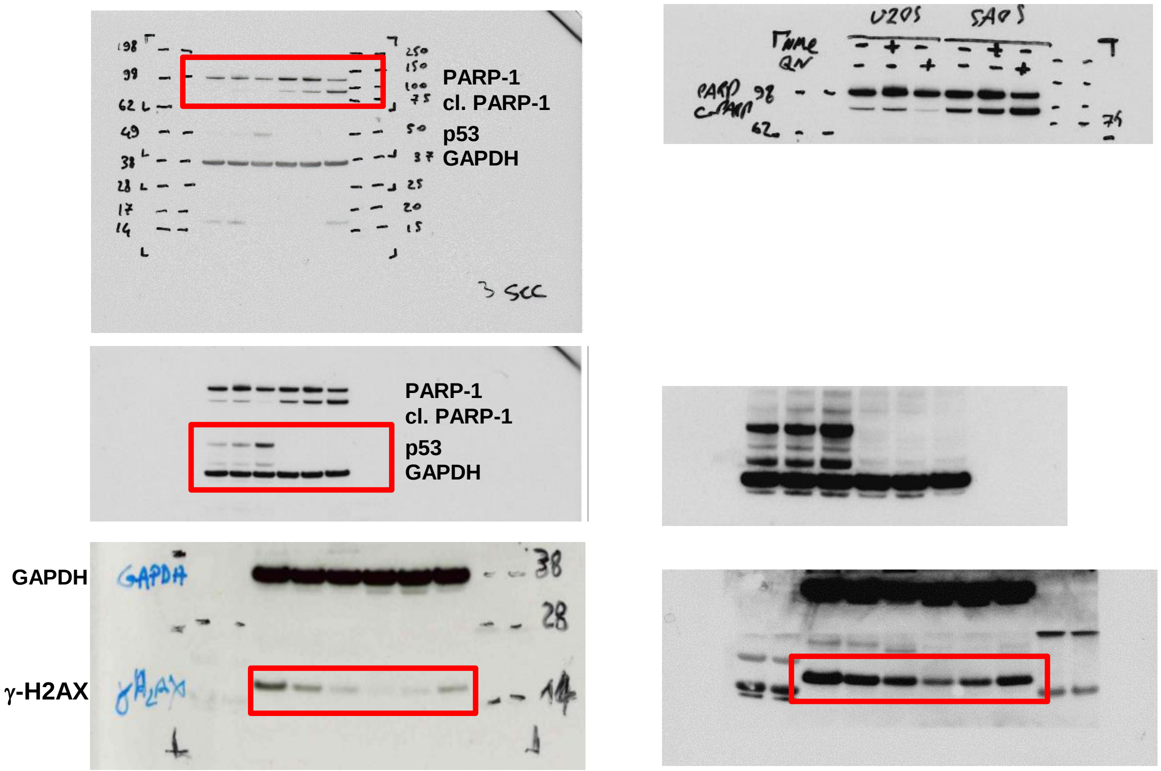

Low exposure

High exposure

FIG 3A

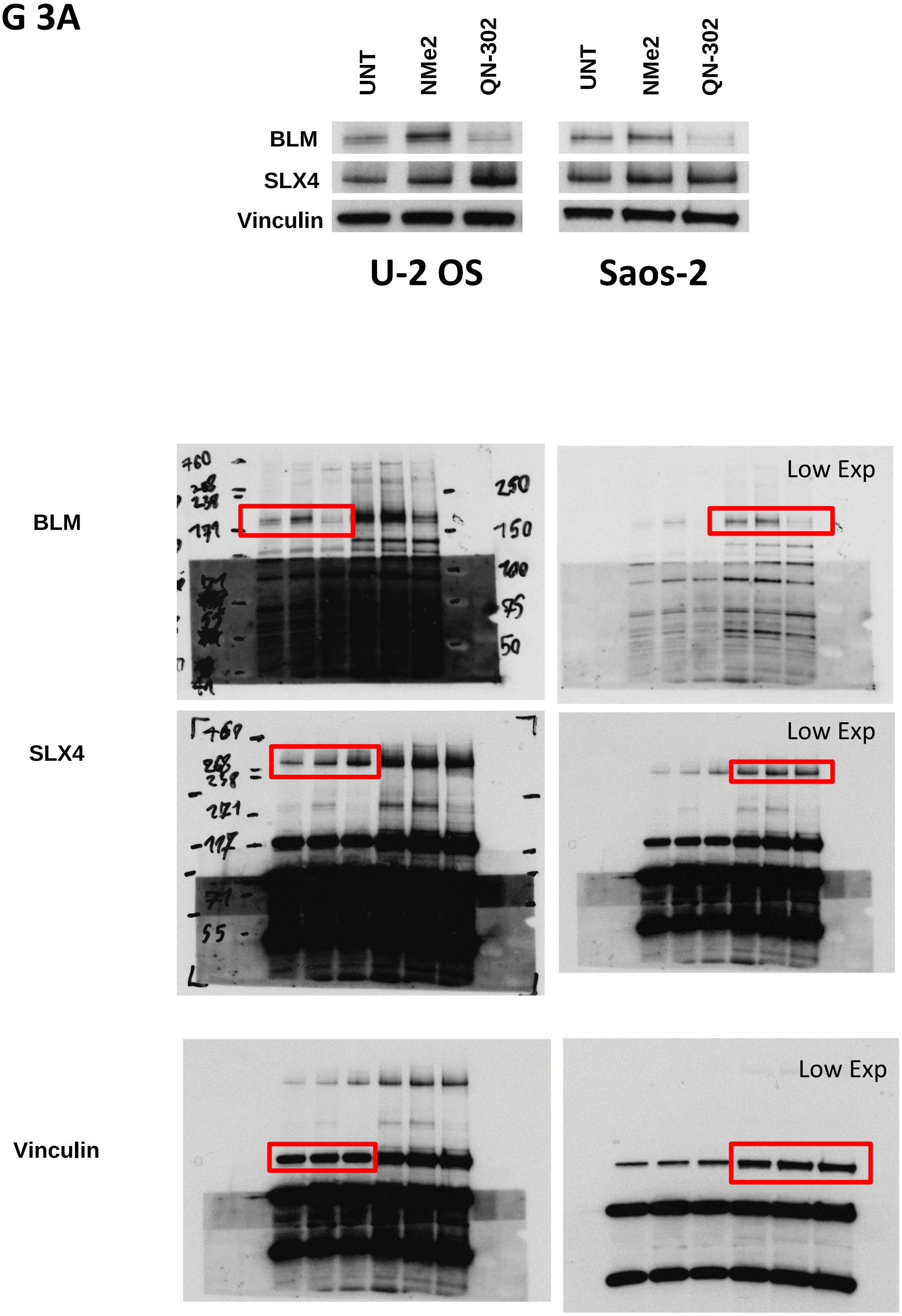
UNT
NMe2
QN-302
UNT
NMe2
QN-302
BLM
SLX4
Vinculin
U-2 OS
Saos-2
BLM
Low Exp
SLX4
Low Exp
Vinculin
Low Exp

FIG 3A

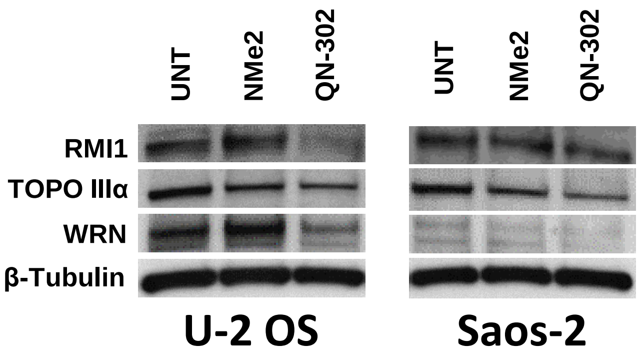

RMI1

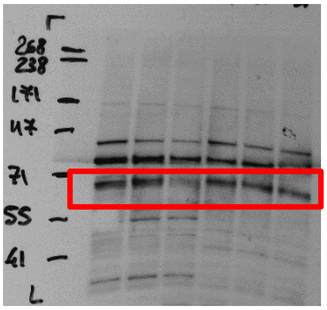

TOPO IIIα

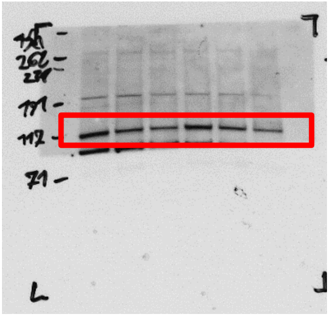

WRN

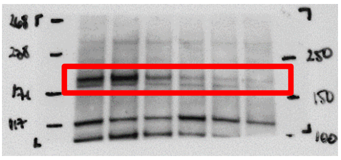

B-Tubulin

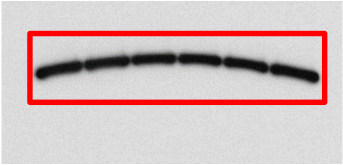

FIG 3A

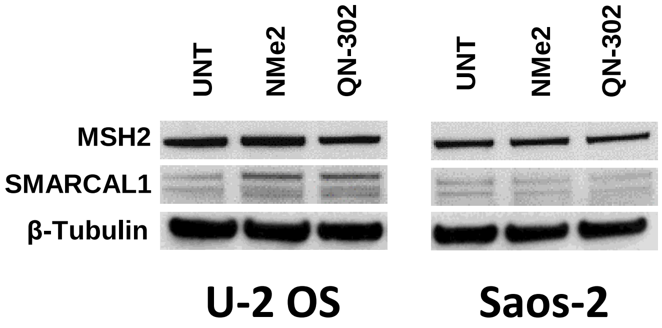
UNT
NMe2
QN-302
UNT
NMe2
QN-302
MSH2
SMARCAL1
β-Tubulin
U-2 OS
Saos-2

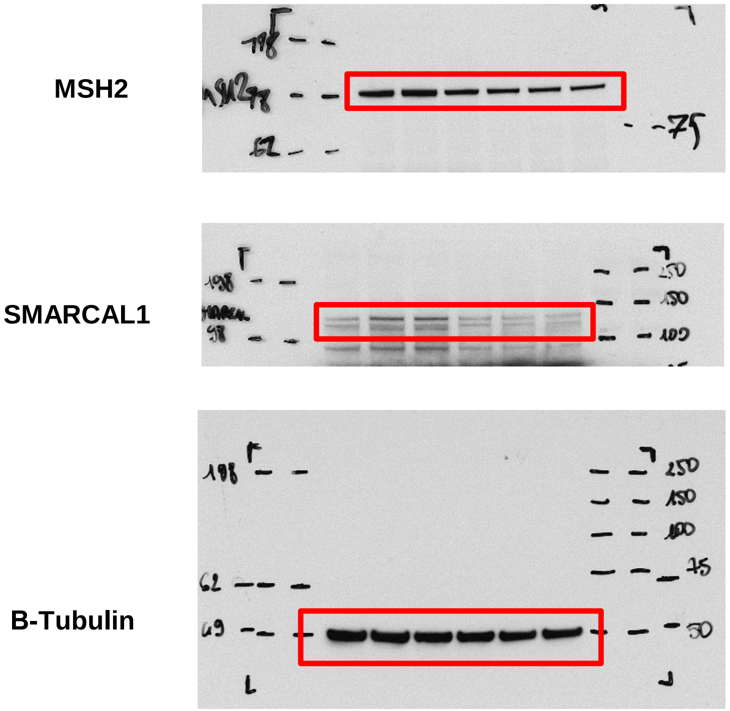
MSH2
SMARCAL1
B-Tubulin

FIG 3B

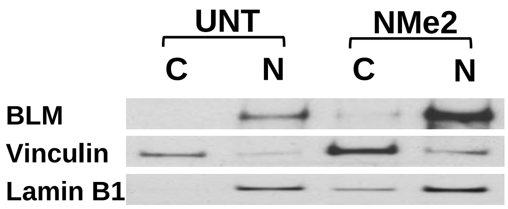
UNT
NMe2
C
N
C
N
BLM
Vinculin
Lamin B1

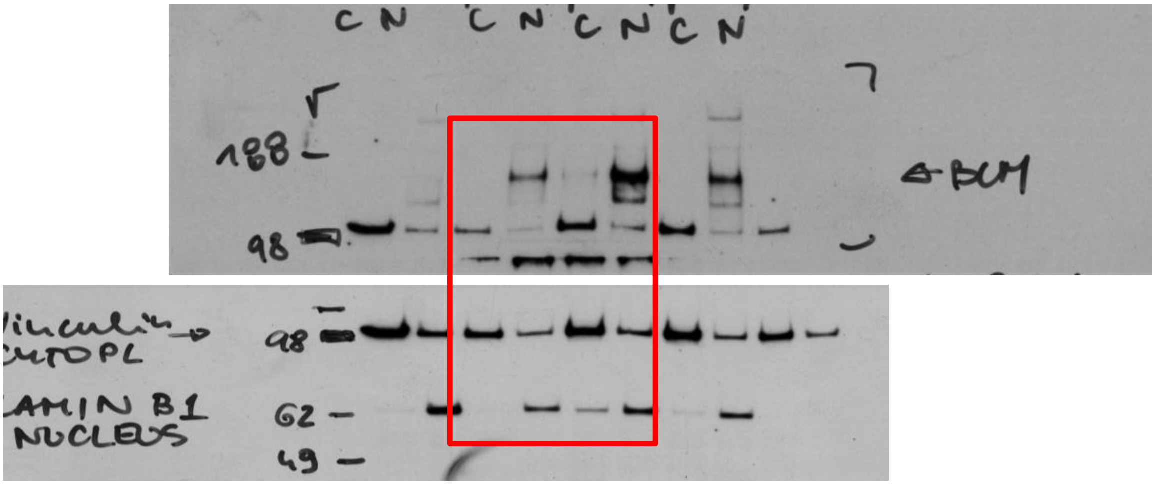
C N C N C N C N
188
98
BLM
98
62
49
CYTOPL
LAMIN B1
NUCLEUS

FIG 3D

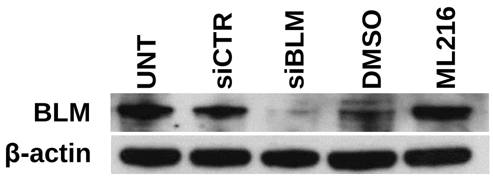
UNT
siCTR
siBLM
DMSO
ML216
BLM
β-actin

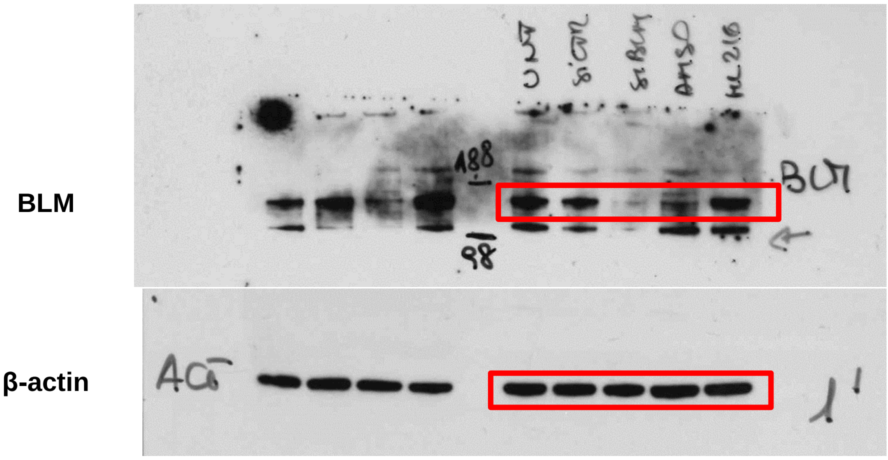
BLM
β-actin

FIG 3F

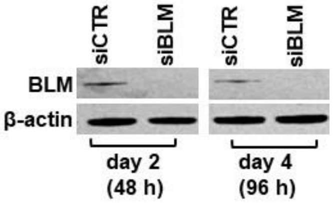
siCTR
siBLM
siCTR
siBLM
BLM
β-actin
day 2
(48 h)
day 4
(96 h)

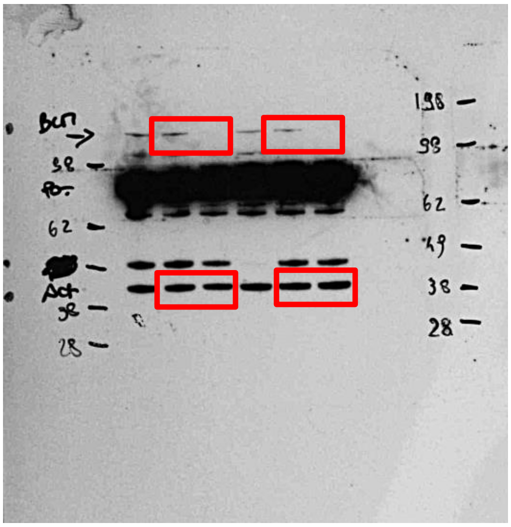

Fig 4C

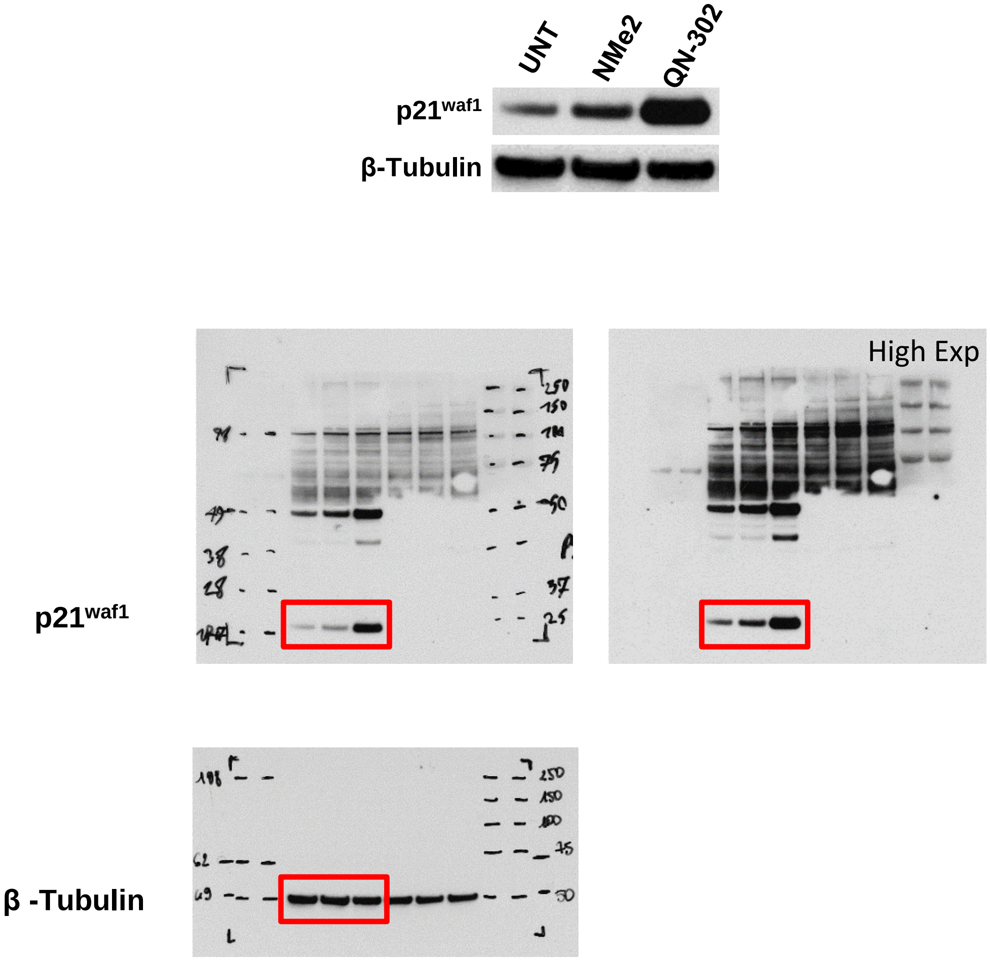

Fig 4G

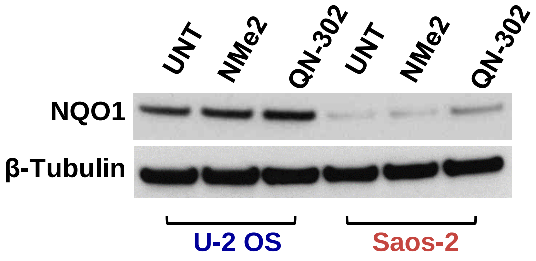

NQO1

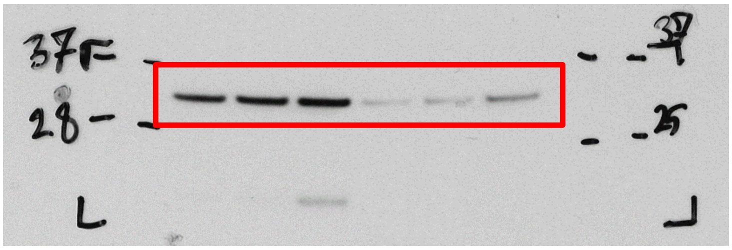

β-Tubulin

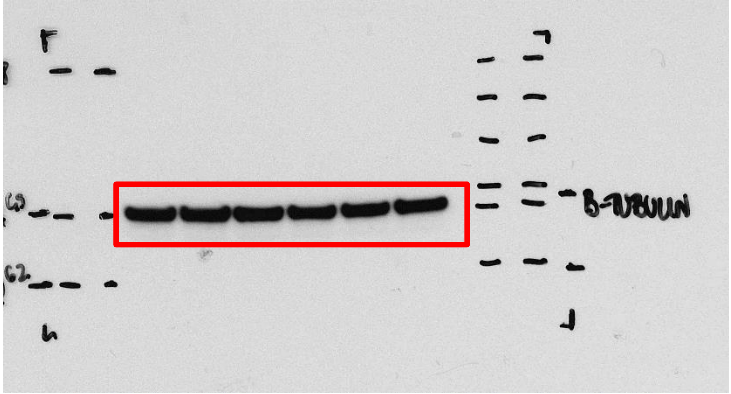

FIG S2A

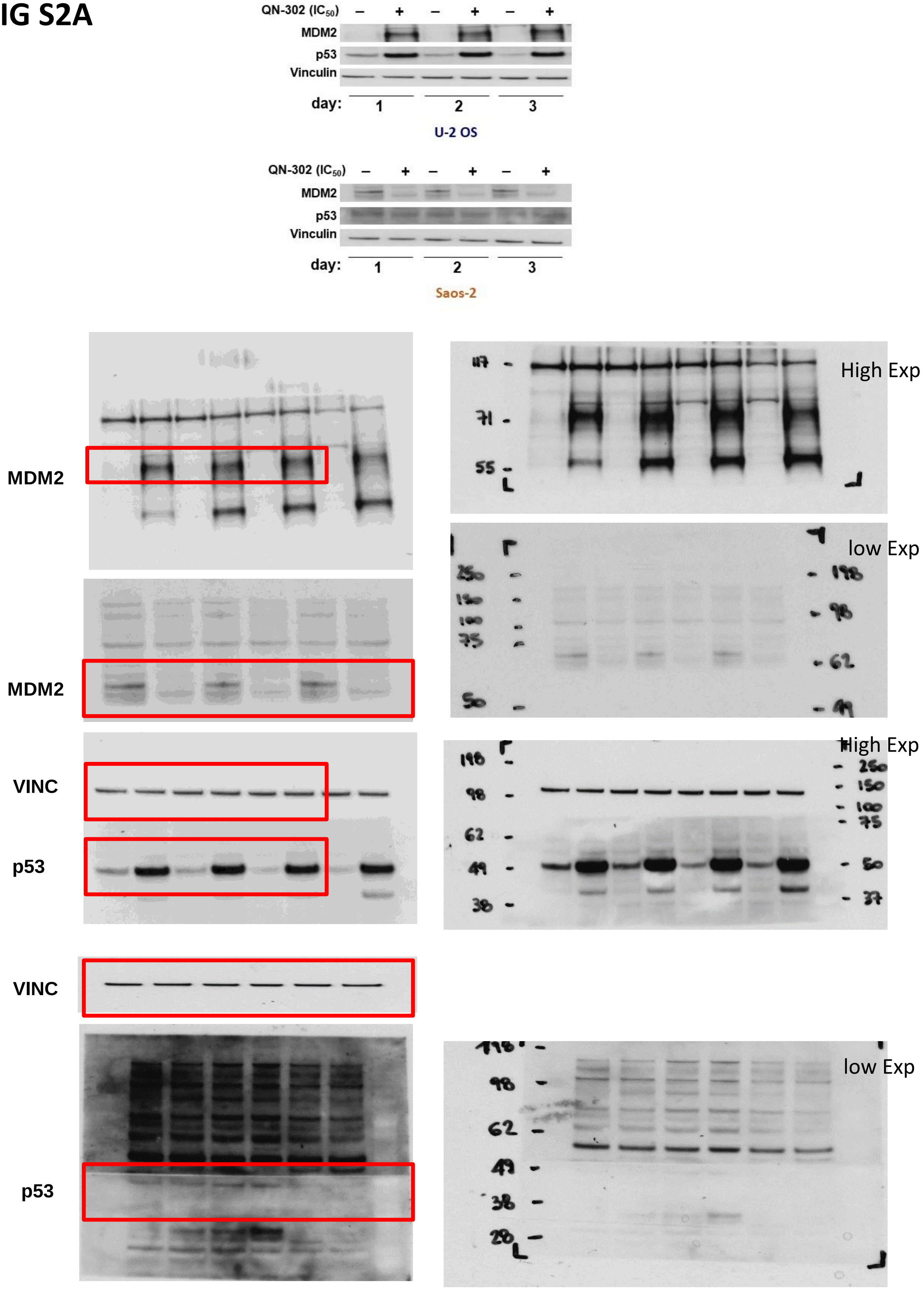
QN-302 (IC_{50}) – + – + – +
MDM2
p53
Vinculin
day: 1 2 3
U-2 OS
QN-302 (IC_{50}) – + – + – +
MDM2
p53
Vinculin
day: 1 2 3
Saos-2
MDM2
MDM2
VINC
p53
VINC
p53
High Exp
low Exp
High Exp
low Exp

FIG S2B

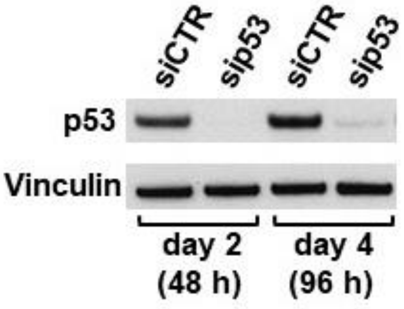
siCTR
sip53
siCTR
sip53
p53
Vinculin
day 2
(48 h)
day 4
(96 h)

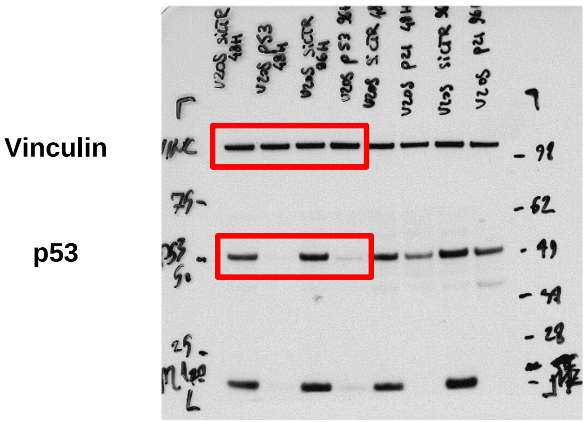
Vinculin
p53

FIG S5C

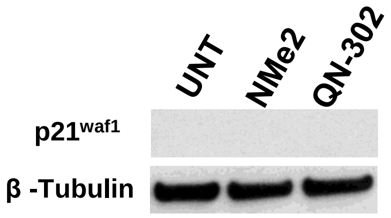

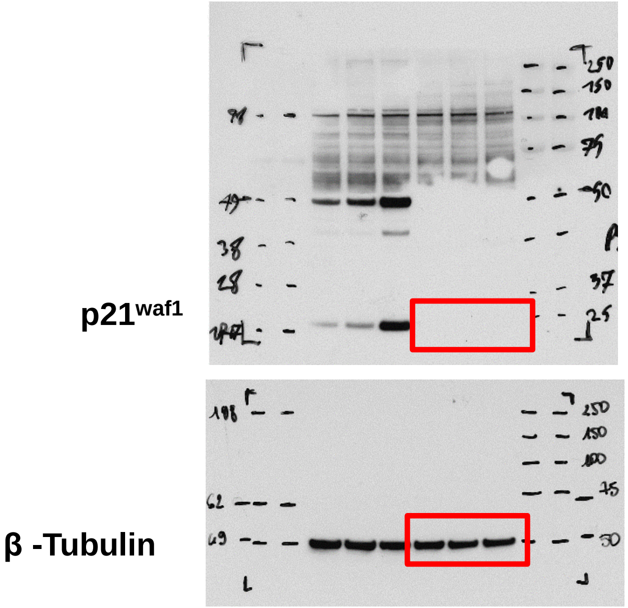

FIG S5D

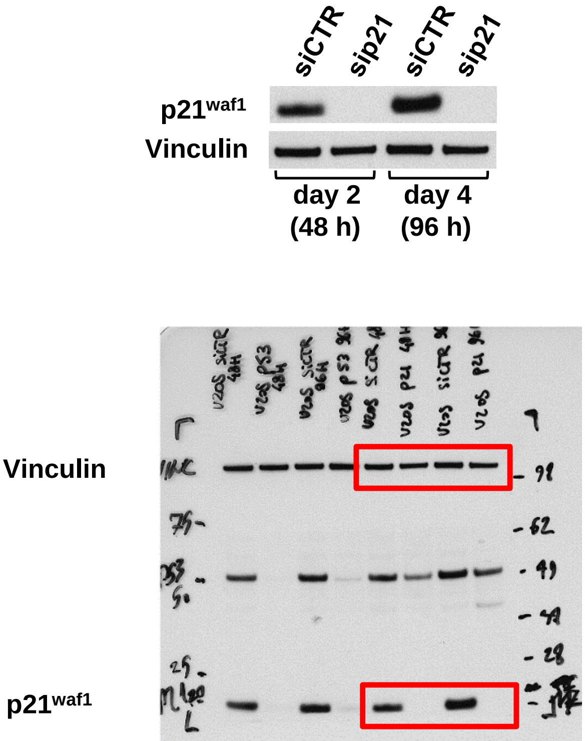
siCTR
sip21
siCTR
sip21
p21waf1
Vinculin
day 2
(48 h)
day 4
(96 h)
Vinculin
p21waf1

FIG S6C

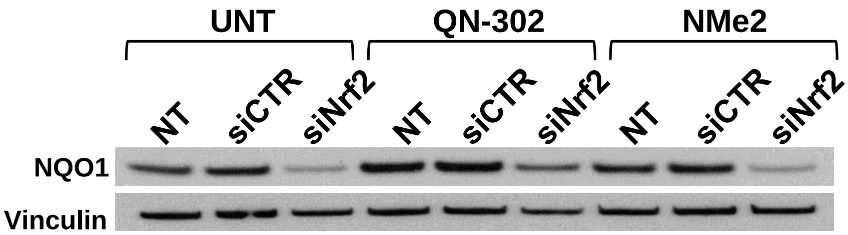

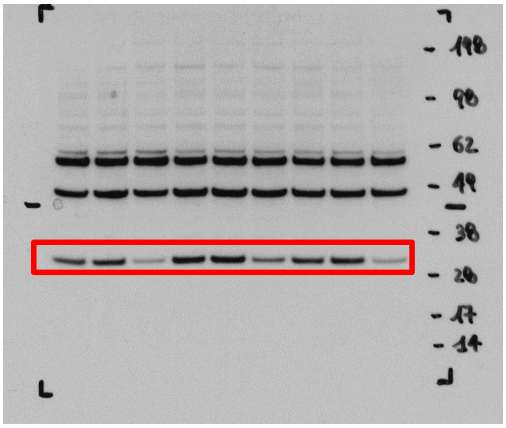

NQO1

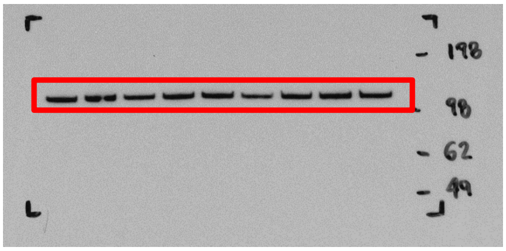

Vinculin

FIG S6D

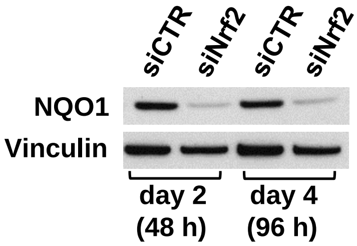
siCTR
siNrf2
siCTR
siNrf2
NQO1
Vinculin
day 2
(48 h)
day 4
(96 h)

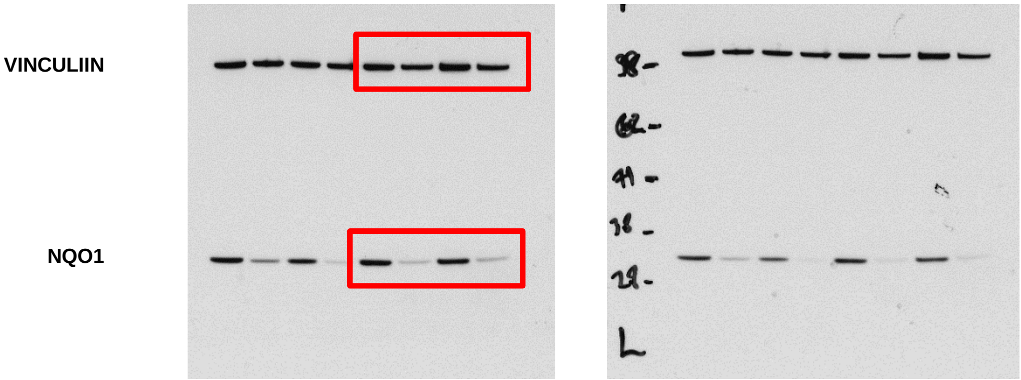
VINCULIIN
NQO1
